# Mailed Outreach for Colorectal Cancer Screening in Community Health Centers: The CARES Pragmatic Cluster Randomized Clinical Trial

**DOI:** 10.1001/jamainternmed.2026.1170

**Published:** 2026-04-27

**Authors:** Folasade P. May, Suzanne Brodney, Jessica J. Tuan, Sapna Syngal, Andrew T. Chan, Beth Glenn, Gina Johnson, Yuchiao Chang, David A. Drew, Beverly Moy, Nicolette J. Rodriguez, Erica T. Warner, Adjoa Anyane-Yeboa, Chinedu Ukaegbu, Anjelica Q. Davis, Kimberly Schoolcraft, Susan Regan, Kelley Le Beaux, Ellen T. Lee, Roopa Bhat, Alexis Gordon, Linh K. Phan, Andrea Fernanda Cortés Chirino, Caylin J. Marotta, Rachel G. Z. Kindermann, Jennifer S. Haas

**Affiliations:** 1 Department of Medicine, David Geffen School of Medicine, UCLA Ronald Reagan Medical Center, University of California, Los Angeles; 2 Vatche and Tamar Manoukian Division of Digestive Diseases, Department of Medicine, David Geffen School of Medicine, Center for Health Sciences, University of California, Los Angeles; 3 Greater Los Angeles Veterans Affairs Healthcare System, Los Angeles, California; 4 UCLA Kaiser Permanente Center for Health Equity, Jonsson Comprehensive Cancer Center, Center for Health Sciences, Los Angeles, California; 5 Division of General Internal Medicine, Massachusetts General Hospital, Boston; 6 Division of Gastroenterology, Brigham and Women’s Hospital, Boston, Massachusetts; 7 Population Sciences and Cancer Genetics and Prevention Divisions, Dana Farber Cancer Institute, Boston, Massachusetts; 8 Harvard Medical School, Boston, Massachusetts; 9 Clinical and Translational Epidemiology Unit, Massachusetts General Hospital and Harvard Medical School, Boston; 10 Division of Gastroenterology, Massachusetts General Hospital and Harvard Medical School, Boston; 11 Broad Institute of Massachusetts Institute of Technology and Harvard, Cambridge; 12 Channing Division of Network Medicine, Department of Medicine, Brigham and Women’s Hospital, and Harvard Medical School, Boston, Massachusetts; 13 Department of Immunology and Infectious Diseases, Harvard T.H. Chan School of Public Health, Boston, Massachusetts; 14 Department of Health Policy and Management, UCLA Fielding School of Public Health, Los Angeles, California; 15 UCLA Center for Cancer Prevention and Control Research, UCLA Jonsson Comprehensive Cancer Center, UCLA School of Public Health, Los Angeles, California; 16 Health Promotion Disease Prevention Programs, Great Plains Tribal Leaders Health Board, Rapid City, South Dakota; 17 Massachusetts General Brigham Cancer Institute, Mass General Brigham, Harvard Medical School, Boston; 18 Mongan Institute, Massachusetts General Hospital and Harvard Medical School, Boston; 19 Fight Colorectal Cancer, Springfield, Missouri; 20 St John’s Community Health, Los Angeles, California

## Abstract

**Importance:**

Colorectal cancer (CRC) is the second most common cause of cancer mortality in the US and disproportionately impacts individuals in underresourced settings.

**Objective:**

To compare 2 mailed population outreach approaches to increase CRC screening uptake among screening-eligible adults in community health centers (CHCs).

**Design, Setting, and Participants:**

This pragmatic cluster randomized clinical trial was conducted in 8 CHCs and an additional site in a nonrandomized parallel protocol. The CHCs were located in the greater Boston area in Massachusetts and Los Angeles County in California (randomized sites), and Rapid City, South Dakota (parallel site). Patients were enrolled in the trial between June 7, 2023, and October 24, 2023. English- or Spanish-speaking primary care patients aged 45 to 75 years, who were due for CRC screening, were eligible to participate.

**Interventions:**

Patients received either mailed fecal immunochemical test (FIT) with automated text message outreach from study personnel or mailed FIT-DNA with the manufacturer’s outreach protocol. Participants in Boston and Los Angeles (randomized sites) with an abnormal FIT or FIT-DNA result were offered standardized navigation to colonoscopy.

**Main Outcomes and Measures:**

The primary outcome was CRC screening participation using any modality (FIT, FIT-DNA, or colonoscopy) within 90 days. Secondary outcomes were screening within 180 days and time to screening participation. The completion of follow-up colonoscopy within 180 days of an abnormal stool test result was also studied.

**Results:**

Among 5127 participants in the RCT regions, 2435 (47.5%) were in the FIT group, and 2692 (52.5%) were in the FIT-DNA group. The mean (SD) age was 54.5 (8.1) years; 3018 (58.9%) were female, and 2109 (41.1%) were male. There were 3818 Hispanic individuals (74.5%), 369 non-Hispanic Black individuals (7.2%), 763 non-Hispanic White individuals (14.9%), and 58 individuals of another race (1.1%). A total of 3363 individuals (65.6%) preferred the Spanish language; 2540 (49.5%) were Medicaid insured, and 614 were (12.0%) uninsured. Screening participation was significantly higher in the FIT-DNA group vs the FIT group at 90 days (751 of 2692 [27.9%] vs 550 of 2435 [22.6%], respectively; *P* = .02) and 180 days (854 of 2692 [31.7%] vs 649 of 2435 [26.7%], respectively). In Boston, screening participation at 90 days was higher (628 of 2208 [28.4%]) than in Los Angeles (673 of 2919 [23.1%]). Findings were similar at 180 days. Among the 100 individuals with an abnormal stool test result, 36 (36.0%) completed a colonoscopy within 180 days.

**Conclusions and Relevance:**

In this cluster randomized clinical trial, CRC screening uptake was higher in the FIT-DNA group than in the FIT group and was higher in Boston compared to Los Angeles CHCs. The follow-up colonoscopy rate within 6 months was suboptimal, even with the availability of navigation.

**Trial Registration:**

ClinicalTrials.gov Identifier: NCT05714644

## Key Points

**Question:**

What is the most effective mailed population outreach approach (fecal immunochemical test [FIT] or FIT-DNA) to increase colorectal cancer screening uptake among screening-eligible adults who receive health care in community health centers (CHCs)?

**Findings:**

In this pragmatic cluster randomized clinical trial of 5127 patients, screening participation was significantly higher in CHCs randomized to FIT-DNA than in CHCs randomized to mailed FIT outreach at 90 days (27.9% vs 22.6%) and at 180 days (31.7% vs 26.7%).

**Meaning:**

FIT-DNA may be a more advantageous approach to increase colorectal cancer screening uptake compared to mailed FIT outreach in CHCs.

## Teaser

This cluster randomized clinical trial compares mailed outreach approaches to increase colorectal cancer screening uptake among adults in community health centers who are due for screening.

## Introduction

Colorectal cancer (CRC) is the second most common cause of cancer mortality in the US and disproportionately affects individuals in underresourced settings.^1,2^ CRC screening can reduce incidence and mortality but is underused, particularly in community health centers (CHCs) that deliver primary care to safety-net populations.^3,4^ CRC screening use in CHCs is below the national average due to patient (eg, limited health literacy), clinician (eg, lack of recommendation), and health system factors (eg, resource constraints).^5,6,7,8,9^

Stool-based screening tests are common in CHCs where access to colonoscopy is limited. Fecal immunochemical test (FIT) is an inexpensive, annual CRC screening modality widely used in CHCs. Although FIT is typically provided during a visit, there is increasing evidence for mailed outreach to increase uptake.^10,11^ FIT-DNA is a newer stool-based screening test, with increasing popularity in CHCs, commonly performed every 3 years and mailed directly to patients by the manufacturer.^12,13^ Both tests require follow-up colonoscopy to complete the screening process if a result is abnormal; however, of those who need follow-up care, the number who actually receive it is low.^14,15,16,17^

Evidence-based interventions to increase CRC screening participation in underserved populations include educational media, reminders, navigation, and mailed outreach.^18,19,20^ In a recent CHC study, patients who received mailed FIT outreach were substantially more likely to complete screening at 6 months than patients who did not (600 of 2001 [30.0%] vs 194 of 2001 [9.7%]; *P* < .001).^10^ FIT-DNA has the potential to improve screening and outcomes in CHCs due to patient preference,^21^ a manufacturer-administered patient assistance program that does not require CHC staff effort,^22^ and higher sensitivity compared to FIT.^3^ Studies comparing the effectiveness of these 2 approaches on screening participation in CHCs are lacking.

## Methods

### Study Design and Participants

This pragmatic, cluster randomized clinical trial (RCT) was approved by the Mass General Brigham Institutional Review Board for the sites in the greater Boston area, Massachusetts, and Los Angeles County, California, and by the Great Plains Area Indian Health Service Institutional Review Board for Rapid City, South Dakota (see Supplement 1 for the trial protocol). To ensure cultural appropriateness in South Dakota, the study protocol and patient materials were reviewed and approved by tribal leaders and community members. The study was considered minimal risk and granted a waiver of written consent.^23^ The Consolidated Standards of Reporting Trials (CONSORT) reporting guideline was followed.

Details of the study design were previously published.^23^ Briefly, the Community Collaboration to Advance Racial/Ethnic Equity in Colorectal Cancer Screening (CARES) study is a pragmatic cluster RCT that compares 2 population outreach approaches to increase CRC screening uptake in CHCs (Figure, A): mailed FIT outreach with automated text reminders and mailed FIT-DNA with the manufacturer’s patient assistance program. We hypothesized that the FIT-DNA group would have higher participation given the centralized patient assistance program and the 3-year screening interval.^24^ Participants were enrolled between June 7, 2023, and October 24, 2023. There were no other organized CRC screening outreach interventions in the CHCs during this period.

**Figure 1.**
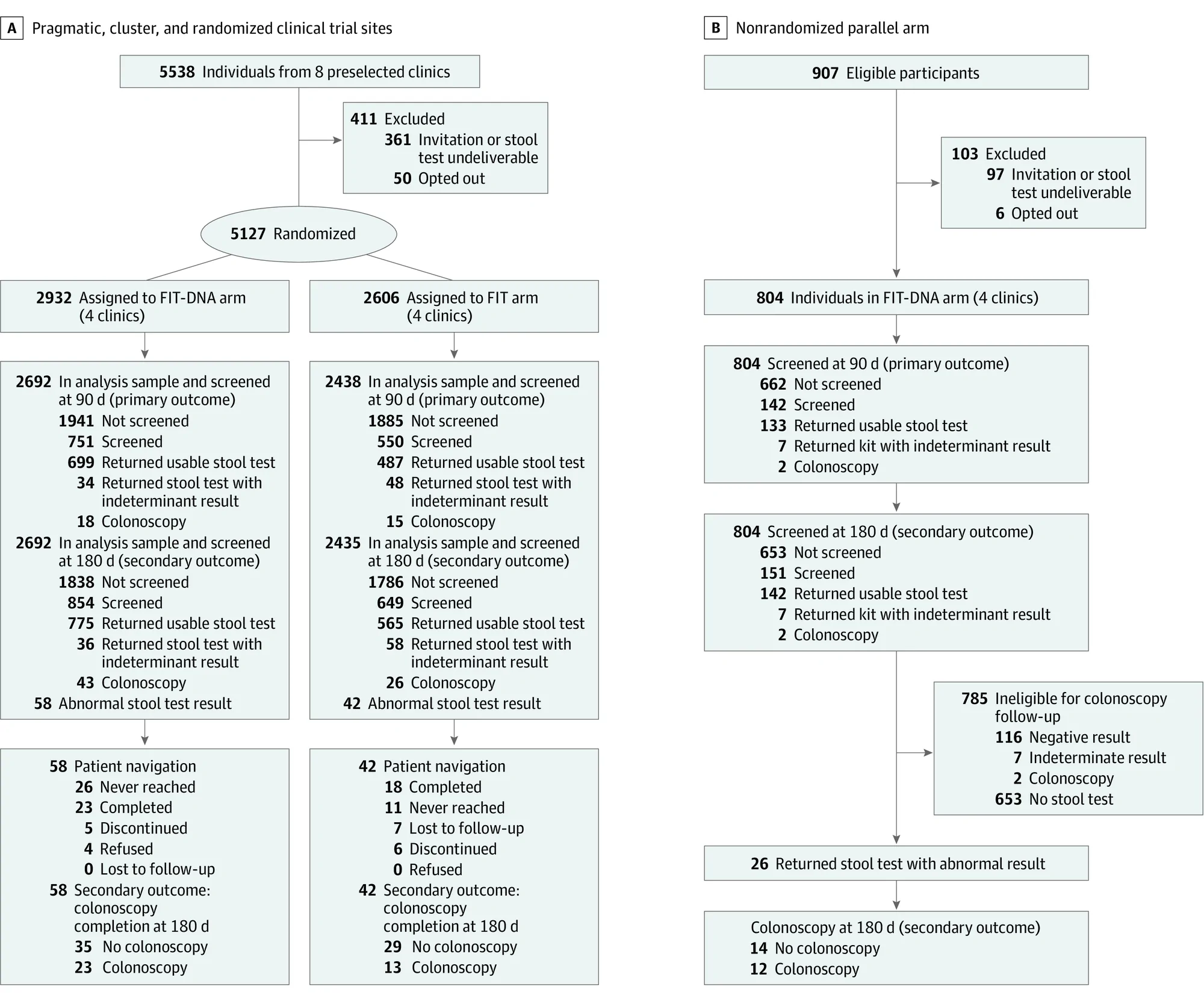
Flow Diagrams of Pragmatic Cluster Randomized Clinical Trial and Parallel Group CRC indicates colorectal cancer; EHR, electronic health record; FIT, fecal immunochemical test.

The RCT included 8 sites: 4 CHC sites in greater Boston (baseline screening rate: 66.5%) and 4 in Los Angeles (baseline screening rate: 23.9%). A site in Rapid City, South Dakota (baseline screening rate: 35.2%) participated in a parallel protocol to receive FIT-DNA but was not randomized as it did not have a comparator (Figure, B).^23^

Individuals were eligible if they were (1) aged 45 to 75 years; (2) due for CRC screening (no FIT or fecal occult blood test in past year, FIT-DNA in prior 3 years, or colonoscopy in past 10 years); (3) received care from a primary care clinician at the CHC^23^; and (4) spoke English or Spanish. We excluded individuals who did not have a physical mailing address or phone number listed in the electronic health record (EHR), had prior documentation of CRC, Crohn disease, ulcerative colitis, or prior abnormal colonoscopy.

### Randomization

In Boston and Los Angeles, we used CHC-level cluster randomization to minimize the chance of contamination within practices. Within each region, we considered the permutations of assigning the 4 CHCs into 2 groups. We chose the permutation with the closest patient volume, racial distribution, and baseline CRC screening participation, then randomly assigned the sites to one of the study groups. Neither patients nor sites were blinded to the allocation.

### Intervention

In both groups, participants received a mailed invitation letter that provided elements of informed consent with the opportunity to opt out. Patients remained under the care of their health care team, who could offer any CRC screening modality. Intervention components were provided in English or Spanish and did not vary by region.

#### FIT-DNA Group

Participants were mailed a FIT-DNA kit from Exact Sciences with instructions on how to complete and return the kit using a standard prepaid mailer. Participants in this group received the standard manufacturer’s patient assistance program, provided with every FIT-DNA test ordered at no additional cost. It includes a combination of scripted telephone calls, text messages, and emails in the users’ preferred language to encourage kit completion.^22^ Exact Sciences collected and provided the authors with data on patient outreach.

#### FIT Group

Patients received a primer text message informing them that a FIT kit would soon arrive at their home address. FIT kits were sent in a separate mailing with a graphic instruction sheet.^25^ Two additional reminder text messages were sent at day 14 and day 28 (1-way texting; content previously published,^23^ English and Spanish). We used the FIT kit brand already in routine use (Boston: Polymedco; Los Angeles and South Dakota: Hemosure iFOB test).

#### Navigation for Patients With Abnormal Results

Participants in Boston and Los Angeles with an abnormal FIT or FIT-DNA result were offered standardized phone navigation^23^ by nonclinical study team members proficient in English or Spanish, to address colonoscopy barriers and encourage completion. In South Dakota, patients with abnormal results received usual follow-up care, which included nonstandardized outreach from clinical staff.

### Outcomes

The primary outcome was CRC screening participation using any screening modality (FIT, FIT-DNA, colonoscopy) within 90 days of screening test mailing. Because participants remained under the care of their clinical team, participants may have been screened using a different modality than assigned by the trial. We therefore examined screening participation by study group (per protocol) and as treated. Screening participation was defined as completion of a test regardless of result. Secondary outcomes included (1) screening participation within 180 days, and (2) time to screening. We also describe colonoscopy completion within 180 days of an abnormal stool test result.

### Statistical Analysis

All sociodemographic, clinical, and outcome data were obtained from CHC EHRs. For the RCT, the primary intent-to-treat analysis (per protocol) included all participants who did not opt out. A secondary analysis used a propensity score–matched sample to ensure balance between the 2 groups on age, gender, race and ethnicity, and insurance status. We also conducted a sensitivity analysis excluding participants who might have completed screening before receiving the mailed screening kit, defined as within 7 days of kit mailing. Separately, we analyzed results for South Dakota participants enrolled to receive FIT-DNA in the parallel group.

We compared the proportions of eligible individuals who participated in screening within 90 or 180 days between study groups using binomial regression models with identity link and the generalized estimating equations to account for the correlated nature of data within CHCs, adjusting for age and race and ethnicity, which were unbalanced between the groups.^23^ We also used generalized estimating equations to examine the dichotomous outcome of colonoscopy completion. We compared time to screening between the groups using a log-rank test. We also explored the heterogeneity of treatment effect by conducting subgroup analyses for prespecified subgroups of interest (region, race and ethnicity, age, and language)^23^ and added insurance subgroups as a post hoc comparison.

Among participants who returned stool tests within 180 days, we compared the distribution of test results between study groups using Fisher exact tests. Among those with an abnormal result, we compared colonoscopy completion within 180 days by group and region using Fisher exact tests and compared time to colonoscopy completion between the 2 groups using a log-rank test. All analyses were conducted using SAS statistical software, version 9.4 (SAS Institute).

### Sample Size and Statistical Power

For the comparison between the FIT-DNA and FIT groups across the 8 randomized sites, we originally anticipated recruiting 5500 individuals. The effective sample size was estimated to be 415 per group, assuming a conservative intraclass correlation coefficient of 0.1 within clinicians and a total of 96 clinicians. This sample size provided greater than 80% power to detect a 10% difference (a clinically meaningful effect size) in screening test completion between the 2 study groups with a 2-sided significance level of .05, assuming any completion in the FIT-DNA group. The data analysis took place between January 2025 and February 2026.

## Results

### Study Population Characteristics

The final sample size was 5127 participants due to a small number of patients who opted out (n = 50) and undeliverable invitations or kits (n = 361). Among 5127 participants in the RCT regions, 2435 were in the FIT group, and 2692 were in the FIT-DNA group. The mean (SD) age was 54.5 (8.1) years; 3018 (58.9%) were female, and 2109 (41.1%) were male. There were 3818 Hispanic individuals (74.5%), 369 non-Hispanic Black individuals (7.2%), 763 non-Hispanic White individuals (14.9%), 58 individuals of another race (3.5%) (including non-Hispanic American Indian, Asian, Pacific Islander, and multiracial), and 119 with missing data. A total of 3363 individuals (65.6%) preferred the Spanish language; 2540 (49.5%) were Medicaid insured, and 614 were (12.0%) uninsured. Demographics varied by region (Table 1). Characteristics by site, including South Dakota, are presented in eTable 1 in Supplement 2.

**Table 1.** Pragmatic Cluster Randomized Clinical Trial Population Demographics Overall and by Community Health Center Region

Characteristic	No. (%)							*P* value^a^
	Total (N = 5127)	FIT group			FIT-DNA group			
		Boston (n = 950)	LA (n = 1485)	Total (n = 2435)	Boston (n = 1258)	LA (n = 1434)	Total (n = 2692)	
Age, mean (SD), y	54.5 (8.1)	53.2 (8.8)	55.9 (7.5)	54.9 (8.1)	52.5 (8.5)	55.8 (7.5)	54.2 (8.1)	.003
Age category, y								
<50	2041 (39.8)	562 (59.2)	348 (23.4)	910 (37.4)	774 (61.5)	357 (24.9)	1131 (42.0)	<.001
≥50	3086 (60.2)	388 (40.8)	1137 (76.6)	1525 (62.6)	484 (38.5)	1077 (75.1)	1561 (58.0)	
Sex								
Female	3018 (58.9)	569 (59.9)	868 (58.5)	1437 (59.0)	727 (57.8)	854 (59.6)	1581 (58.7)	.84
Male	2109 (41.1)	381 (40.1)	617 (41.5)	998 (41.0)	531 (42.2)	580 (40.4)	1111 (41.3)	
Race and ethnicity								
Hispanic	3818 (74.5)	396 (41.7)	1326 (89.3)	1722 (70.7)	774 (61.5)	1322 (92.2)	2096 (77.9)	<.001
Non-Hispanic Black	369 (7.2)	75 (7.9)	121 (8.1)	196 (8.0)	105 (8.3)	68 (4.7)	173 (6.4)	
Non-Hispanic White	763 (14.9)	431 (45.4)	25 (1.7)	456 (18.7)	279 (22.2)	28 (2.0)	307 (11.4)	
Other/unknown^b^	177 (3.5)	48 (5.1)	13 (0.9)	61 (2.5)	100 (7.9)	16 (1.1)	116 (4.3)	
Language								
English	1764 (34.4)	673 (70.8)	234 (15.8)	907 (37.2)	654 (52.0)	203 (14.2)	857 (31.8)	<.001
Spanish	3363 (65.6)	277 (29.2)	1251 (84.2)	1528 (62.8)	604 (48.0)	1231 (85.8)	1835 (68.2)	
Insurance type								
Medicare	441 (8.6)	120 (12.6)	103 (6.9)	223 (9.2)	143 (11.4)	75 (5.2)	218 (8.1)	.17
Medicaid	2540 (49.5)	183 (19.3)	1037 (69.8)	1220 (50.1)	292 (23.2)	1028 (71.7)	1320 (49.0)	
Private	1514 (29.5)	612 (64.4)	70 (4.7)	682 (28.0)	756 (60.1)	76 (5.3)	832 (30.9)	
Uninsured	614 (12.0)	34 (3.6)	268 (18.0)	302 (12.4)	63 (5.0)	249 (17.4)	312 (11.6)	
Other/unknown^c^	18 (0.4)	1 (0.1)	7 (0.5)	8 (0.3)	4 (0.3)	6 (0.4)	10 (0.4)	

Abbreviations: FIT, fecal immunochemical test; LA, Los Angeles County.

^a^ Comparison of FIT-DNA vs FIT.

^b^ Other includes non-Hispanic American Indian (n = 7), Asian (n = 47), Pacific Islander (n = 1), and multiracial (n = 3). Data on race and ethnicity were missing for 119 participants.

^c^ Other includes payers that may not have covered colorectal cancer screening but were listed in the electronic health record as the primary insurer, including vision insurance, workers’ compensation, motor vehicle insurance, family planning, Ryan White HIV/AIDS Program, and country-sponsored or locally sponsored health services.

### Screening Participation by Study Group

For RCT participants, screening participation was significantly higher in the FIT-DNA group than in the FIT group at 90 days (751 of 2692 [27.9%] vs 550 of 2435 [22.6%], respectively; *P* = .02) and 180 days (854 of 2692 [31.7%] vs 649 of 2435 [26.7%], respectively; Table 2). Results were similar in the sensitivity and secondary analyses (eTables 2 and 3 in Supplement 2). Time to screening was shorter in the FIT-DNA group in the primary sample and the propensity-matched sample (eFigures 1 and 2 in Supplement 2). Screening participation for South Dakota is presented in eTable 4 and eFigure 3 in Supplement 2.

**Table 2.** Primary and Secondary Outcomes of Screening Participation for Pragmatic Cluster Randomized Clinical Trial Participants^a^

Outcome	No. (%)		Adjusted difference (95% CI), %^b^
	FIT (n = 2435)	FIT-DNA (n = 2692)	
Primary outcome: screening participation within 90 d	550 (22.6)	751 (27.9)	4.7 (0.8-8.6)
Secondary outcome: screening participation within 180 d	649 (26.7)	854 (31.7)	4.5 (0.5-8.5)

Abbreviation: FIT, fecal immunochemical test.

^a^ Screening participation is defined as a test returned regardless of the result (eg, normal, abnormal, inconclusive).

^b^ From binomial regression models with identity link adjusting for age and race and ethnicity and accounting for clustering within community health centers; intraclass correlation coefficient was 0.004 for primary outcome and 0.003 for secondary outcome.

Outreach in the FIT-DNA group included mailed letters (99.5%), telephone calls (97.4%), text messages (94.0%), and emails (4.8%) (eTable 9 in Supplement 2). Median outreach attempts were 10 overall, 8 for those who completed FIT-DNA, and 10 for those who did not.

In Boston, screening participation at 90 days was significantly higher (628 of 2208 [28.4%]) than in Los Angeles (673 of 2919 [23.1%]; Table 3). In Boston, FIT and FIT-DNA groups had similar screening completion (276 of 950 [29.1%] vs 352 of 1258 [28.0%], respectively). However, in Los Angeles, participation was significantly higher in the FIT-DNA group (FIT: 274 of 1485 [18.5%] vs FIT-DNA: 399 of 1434 [27.8%]). Screening participation at 180 days followed similar patterns.

**Table 3.** Subgroup Analyses of Screening Participation for Pragmatic, Cluster-Randomized Clinical Trial Participants

Subgroup	Participant, No./total No. (%)			Adjusted difference (95% CI), %^a^
	Total	FIT	FIT-DNA	
**Screening participation within 90 d**				
Region				
Boston	628/2208 (28.4)	276/950 (29.1)	352/1258 (28.0)	−0.6 (−5.7 to 4.6)
LA	673/2919 (23.1)	274/1485 (18.5)	399/1434 (27.8)	9.4 (8.3 to 10.6)
Age category, y				
<50	499/2041 (24.4)	217/910 (23.8)	282/1131 (24.9)	1.4 (−2.4 to 5.3)
≥50	802/3086 (26.0)	333/1525 (21.8)	469/1561 (30.0)	6.9 (1.8 to 12.0)
Race and ethnicity				
Hispanic	956/3818 (25.0)	362/1722 (21.0)	594/2096 (28.3)	6.6 (2.4 to 10.8)
Non-Hispanic, Black	74/369 (20.1)	41/196 (20.9)	33/173 (19.1)	−4.7 (−7.9 to −1.6)
Non-Hispanic, White	219/763 (28.7)	127/456 (27.9)	92/307 (30.0)	3.0 (−10.5 to 16.6)
Other/unknown^b^	52/177 (29.4)	20/61 (32.8)	32/116 (27.6)	−4.9 (−16.0 to 6.2)
Language				
English	437/1764 (24.8)	226/907 (24.9)	211/857 (24.6)	1.0 (−4.7 to 6.6)
Spanish	864/3363 (25.7)	324/1528 (21.2)	540/1835 (29.4)	7.5 (3.9 to 11.1)
Insurance type				
Medicare	107/441 (24.3)	52/223 (23.3)	55/218 (25.2)	1.6 (−6.0 to 9.2)
Medicaid	648/2540 (25.5)	258/1220 (21.1)	390/1320 (29.5)	8.9 (7.3 to 10.4)
Private	444/1514 (29.3)	201/682 (29.5)	243/832 (29.2)	0.7 (−4.8 to 6.2)
Uninsured	102/614 (16.6)	39/302 (12.9)	63/312 (20.2)	8.6 (7.2 to 10.0)
**Screening participation within 180 d**				
Region				
Boston	730/2208 (33.1)	316/950 (33.3)	414/1258 (32.9)	0.3 (−5.6 to 6.1)
LA	773/2919 (26.5)	333/1485 (22.4)	440/1434 (30.7)	8.3 (6.4 to 10.2)
Age category, y				
<50	582/2041 (28.5)	258/910 (28.4)	324/1131 (28.6)	0.7 (−4.2 to 5.6)
≥50	921/3086 (29.8)	391/1525 (25.6)	530/1561 (34.0)	7.4 (3.2 to 11.7)
Race and ethnicity				
Hispanic	1105/3818 (28.9)	434/1722 (25.2)	671/2096 (32.0)	6.1 (2.0 to 10.2)
Non-Hispanic, Black	88/369 (23.8)	48/196 (24.5)	40/173 (23.1)	−4.2 (−11.7 to 3.3)
Non-Hispanic, White	252/763 (33.0)	145/456 (31.8)	107/307 (34.9)	2.8 (−11.1 to 16.8)
Other/unknown^b^	58/177 (32.8)	22/61 (36.1)	36/116 (31.0)	−5.3 (−17.7 to 7.2)
Language				
English	511/1764 (29.0)	259/907 (28.6)	252/857 (29.4)	1.6 (−4.8 to 8.0)
Spanish	992/3363 (29.5)	390/1528 (25.5)	602/1835 (32.8)	6.4 (2.5 to 10.2)
Insurance type				
Medicare	126/441 (28.6)	62/223 (27.8)	64/218 (29.4)	0.2 (−6.2 to 6.5)
Medicaid	733/2540 (28.9)	312/1220 (25.6)	421/1320 (31.9)	6.4 (3.1 to 9.7)
Private	523/1514 (34.5)	229 (33.6)	294/832 (35.3)	3.7 (−3.7 to 11.1)
Uninsured	120/614 (19.5)	46 (15.2)	74/312 (23.7)	9.8 (8.3 to 11.2)

Abbreviation: FIT, fecal immunochemical test; LA, Los Angeles County.

^a^ From binomial regression models with identity link, adjusting for region for subgroups other than region and accounting for clustering within community health centers.

^b^ Other includes non-Hispanic American Indian (n = 7), Asian (n = 47), Pacific Islander (n = 1), and multiracial (n = 3). Data on race and ethnicity were missing for 119 participants.

### Screening Participation by Subgroups

Higher screening participation was observed in the FIT-DNA vs the FIT group among individuals who were older (aged ≥50 years: 469 of 1561 [30.0%] vs 333 of 1525 [21.8%], respectively), Hispanic (594 of 2096 [28.3%] vs 362 of 1722 [21.0%], respectively), Spanish-speaking (540 of 1835 [29.4%] vs 324 of 1528 [21.2%], respectively), on Medicaid (648 of 2540 [29.5%] vs 258 of 1220 [21.1%], respectively), or uninsured (63 of 312 [20.2%] vs 39 of 302 [12.9%], respectively) (Table 3).

### Abnormal Screening Results by Study Group (Per Protocol) vs As Treated

Among screened individuals in the randomized sites, 100 of 1435 (7.0%) had an abnormal result (FIT group: 42 of 623 [6.7%], FIT-DNA group: 58 of 812 [7.1%]; Table 4). Among individuals randomized to FIT, 7 of 623 (1.1%) completed FIT-DNA, and among those randomized to FIT-DNA, 114 of 812 (14.0%) completed FIT. In the as-treated analysis, the proportion with an abnormality was 50 of 730 (6.8%) and 50 of 705 (7.1%) in the FIT and FIT-DNA groups, respectively. Notably, crossover from FIT-DNA to FIT was more likely as FIT kits are often readily available compared to FIT-DNA tests, which require a clinician’s order.

**Table 4.** Abnormal Screening Results by Study Group Per Protocol vs As Treated

	Total	Per protocol		Rate difference (95% CI), %
		FIT	FIT-DNA	
**Screening test completed**				
No.	n = 1435	n = 623	n = 812	
FIT	730	616	114	
FIT-DNA	705	7	698	
**Per protocol**				
No.	n = 1435	n = 623	n = 812	
Normal	1240 (86.4)	523 (83.9)	717 (88.3)	4.4 (0.7 to 8.0)
Abnormal	100 (7.0)	42 (6.7)	58 (7.1)	0.4 (−2.3 to 3.1)
Inconclusive	95 (6.6)	58 (9.3)	37 (4.6)	−4.8 (−7.5 to −2.1)
**As treated**				
No.	n = 1435	n = 730	n = 705	
Normal	1240 (86.4)	621 (85.1)	619 (87.8)	2.7 (−0.8 to 6.3)
Abnormal	100 (7.0)	50 (6.8)	50 (7.1)	0.2 (−2.4 to 2.9)
Inconclusive	95 (6.6)	59 (8.1)	36 (5.1)	−3.0 (−5.5 to −0.4)

Abbreviation: FIT, fecal immunochemical test.

In Boston, the proportions with an abnormal FIT and FIT-DNA were 6 of 291 (2.1%) and 27 of 372 (7.3%) based on assigned group, and 7 of 312 (2.2%) and 26 of 351 (7.4%), based on the test completed, respectively (eTable 5 in Supplement 2). In Los Angeles, these proportions were 36 of 332 (10.8%) for FIT and 31 of 354 (7.0%) for FIT-DNA (by assigned group), and 43 of 418 (10.3%) for FIT and 24 of 354 (6.8%) for FIT-DNA (by test completed). In South Dakota, the proportion with an abnormal FIT-DNA was 26 of 149 (17.4%) (assigned) and 15 of 130 (11.5%) (test completed).

### Colonoscopy After an Abnormal Test Result

Standardized navigation was attempted for all 100 patients with an abnormal result (Boston: 33, Los Angeles: 67): 37 were never reached, 4 refused navigation, and 59 received navigation (eTable 6 in Supplement 2). Among these 100 patients, 36 completed colonoscopy within 180 days. Colonoscopy completion was similar in the 2 groups (FIT-DNA: 23 of 58 [39.7%], FIT: 13 of 42 [31.0%]; eTable 7 in Supplement 2) as was time to colonoscopy (eFigure 4 in Supplement 2). Boston had higher colonoscopy completion (23 of 33 [69.7%]) than Los Angeles (13 of 67 [19.4%]; eTable 8 in Supplement 2). Colonoscopy data for South Dakota are presented in eTable 8 in Supplement 2.

## Discussion

In this pragmatic cluster RCT in CHCs, we found that CRC screening participation was significantly higher and time to screening shorter with FIT-DNA than with mailed FIT outreach. In Boston, overall screening participation was significantly higher than in Los Angeles, and screening participation in the 2 study groups was similar. For patients with an abnormal FIT or FIT-DNA result, colonoscopic follow-up at 180 days was suboptimal in both groups, though varied by region. Our findings are novel as this is one of the first studies to examine the implementation of FIT-DNA in CHCs and to directly compare the effectiveness of FIT-DNA to mailed FIT outreach in this setting. The STOP CRC and SCORE trials implemented mailed FIT outreach in CHCs and found a 3.4% and 20.3% increase in screening, respectively, compared to usual care.^10,19,26^

We hypothesize that screening participation with FIT-DNA was higher for several reasons. First, the manufacturer’s patient assistance program offered greater support than mailed FIT with automated reminders. Automated texting for mailed FIT outreach^27^ reflects the maximal level of support often feasible in CHCs.^22^ Second, patients presented with the FIT-DNA option may have been motivated by the longer screening interval compared to FIT^21^ or other features of the test. FIT-DNA completion in this study was lower than the manufacturer’s average (71% by 28 days), likely underscoring persistent social and economic barriers to screening for safety-net populations^24^ and clinician influence, as FIT-DNA is typically ordered by clinicians after a discussion with the patient.

The overall difference between FIT-DNA and FIT participation was driven by Los Angeles CHCs, where FIT-DNA uptake was notably higher than FIT. This finding may reflect differences in patient characteristics and prior FIT-DNA offering in the 2 regions. There was a higher prevalence of Spanish-speaking patients in Los Angeles, and this population may be more responsive to the increased FIT-DNA outreach. FIT-DNA was previously available in Boston CHCs but offered for the first time in Los Angeles CHCs through this study. This novelty may have disproportionately motivated some patients.

Despite the high uptake of FIT-DNA in Los Angeles, overall screening participation was higher in Boston. This regional variance is likely attributable to differences in patient populations. In Los Angeles, the study population was largely Hispanic, Spanish-speaking, and uninsured, factors associated with low CRC screening participation.^4,28,29,30,31^ Conversely, Massachusetts offers broad insurance coverage.^32,33^ Our findings emphasize the importance of considering regional and demographic factors when implementing similar interventions.

Our study does not assess cost. From a patient perspective, both FIT and FIT-DNA are covered by insurance, with equivalent low out-of-pocket cost for patients with coverage.^13^ Without insurance, FIT costs vary but can be purchased for approximately $20 online.^34^ FIT-DNA is currently $599.^35^ From the clinic perspective, cost estimates of organized FIT outreach in safety net settings are $22 to $91 per patient screened,^36,37^ depending on outreach approach. Clinics bear the cost of mailing kits (including those not returned) and any staff outreach.

Stool-based CRC screening is only effective if patients with an abnormal result undergo timely colonoscopy.^16,38,39^ With navigation, just over one-third of patients with an abnormal screening result completed colonoscopy within 180 days, higher than prior studies.^10,17,40,41^ Notably, colonoscopy completion was higher in Boston than Los Angeles, likely reflecting the ability of Boston CHCs to refer patients directly to academic health system partners, whereas Los Angeles CHCs referred to outside private and safety-net facilities for colonoscopy.^5,14,28,29,30,31,42,43,44,45^

We include data for a tribal site in South Dakota using a parallel, nonrandomized protocol. Because American Indian individuals have the highest incidence of CRC and the lowest screening participation,^1,2^ these descriptive data provide important insights into conducting culturally sensitive CRC screening research in Native American settings. Mailed FIT-DNA outreach in South Dakota was associated with an increase in participation, with a high proportion of abnormal FIT-DNA results, likely related to low baseline screening participation.

### Strengths and Limitations

This study has many strengths. First, this trial compared the feasibility and effectiveness of mailed FIT and FIT-DNA screening approaches in CHCs to reflect how these strategies could function in routine practice. Mailed outreach is infrequently used in clinical practice in CHCs; thus, our study is an early example of mailed FIT and FIT-DNA implementation in CHCs. As interest in FIT-DNA grows due to increasing insurance coverage, advantageous test characteristics, and the ability to have an outside organization provide the patient support, our study provides insight into the potential benefits of FIT-DNA in these settings. Second, we offered CRC screening to more than 6000 underserved individuals (most were Hispanic, and 14% were American Indian) in 3 regions, a cohort size rarely achieved in CHC CRC screening studies. Third, we demonstrate that with both strategies, most screening was completed in the first 90 days. Fourth, our study emphasizes the need for novel interventions to ensure colonoscopy is accessible after abnormal screening in resource-limited settings.

Our study has limitations. First, CHCs in Boston and Los Angeles used different FIT kit brands,^46,47,48^ which may have contributed to a higher proportion of abnormal results in Los Angeles. This finding highlights the importance of considering FIT performance when selecting a FIT for routine use, given that tests with higher false-positive results will increase the number of patients who need follow-up colonoscopies.^46,49^ Second, we did not include a usual care group, which was not considered acceptable to our clinic partners, given low baseline screening participation. Third, we cannot comment on cost-effectiveness, though FIT-DNA’s 3-year screening interval, higher sensitivity for advanced adenomas, and higher colonoscopy follow-up in some studies may have long-term cost-saving implications important for safety-net settings.^50,51^ Fourth, while randomization at the clinic level minimized the likelihood of contamination, there was imbalance in patient demographics across the 2 groups; our secondary propensity score-matched sample analysis allowed us to control for these differences. Fifth, we could not randomize the South Dakota site. Nonetheless, including South Dakota in a parallel design was important to obtain data about an understudied setting and population. Finally, we cannot disentangle the role of follow-up intensity and test type on outcomes.

## Conclusions

This cluster RCT is an early demonstration of mailed FIT outreach and FIT-DNA implementation in CHCs. FIT-DNA emerged as the more advantageous approach to increase screening uptake. While overall participation was modest, our findings are clinically important and inform future practice. Given that CRC outcomes are often poorest among underserved populations receiving care in CHCs, evidence-based interventions to improve outcomes must be research and policy priorities.

## Notes

Supplement 1. Trial Protocol and Statistical Analysis Plan

Supplement 2. eFigure 1. Time to screening participation over 180 days by study arm, among pragmatic, cluster randomized clinical trial participants (Boston and Los Angeles) eFigure 2. Time to screening participation by study arm from propensity score-matched sample of pragmatic, cluster randomized clinical trial participants (Boston and Los Angeles) eFigure 3. Time to screening participation among South Dakota participants (parallel arm) eFigure 4. Time to follow-up colonoscopy completion over 180 days among pragmatic, cluster randomized clinical trial participants (Boston and Los Angeles) with abnormal stool test results eTable 1. Population characteristics by site, including South Dakota eTable 2. Sensitivity analysis excluding participants who might have completed screening prior to receiving the screening kit among pragmatic, cluster randomized clinical trial participants (Boston and Los Angeles) eTable 3. Secondary analysis using the propensity score matched sample among pragmatic, cluster randomized clinical trial participants (Boston and Los Angeles) eTable 4. Primary and secondary outcomes of screening participation of South Dakota participants (parallel arm) eTable 5. Abnormal Screening Results by Study Arm (Per Protocol) vs. As Treated within 180 days, by region eTable 6. Navigation outcomes for patients with a positive stool test result (randomized clinical trial participants) eTable 7. Colonoscopy completion within 180 days after an abnormal stool test, pragmatic, cluster randomized clinical trial sites only eTable 8. Colonoscopy completion within 180 days after an abnormal stool test by region eTable 9. Exact Sciences Patient Navigation Outreach Data (within 45 days of FIT-DNA kit mailing)

Supplement 3. Data Sharing Statement
