# Supplementary material for: Mailed Outreach for Colorectal Cancer Screening in Community Health Centers: The CARES Pragmatic Cluster Randomized Clinical Trial
Source: JAMA Intern Med. 2026 Apr 27;186(6):703–12. doi: 10.1001/jamainternmed.2026.1170 (PMC13122492; doi:10.1001/jamainternmed.2026.1170)
Supplement: Supplement 1. — Trial Protocol and Statistical Analysis Plan [file jamainternmed-e261170-s001.pdf]

Mass General Brigham

Institutional Review Board
Intervention/Interaction Detailed Protocol

Principal Investigator:	Jennifer Haas, MD
Project Title:	Community Collaboration to Advance Racial/Ethnic Equity in CRC Screening (CARES):
Version Date:	March 7, 2024

---

1. Background and Significance

There is a high public health burden of colorectal cancer (CRC). CRC is the second most common cause of cancer-related deaths in the United States (US).[1] Each year, over 145,000 Americans are told they have CRC, and more than 53,000 die from this disease.[2] Black Americans have the highest incidence of CRC (45.7 per 100,000), followed by American Indians/Alaska Natives (AI/AN – 43.3), Whites (38.6), Latino/a/x (referred to as Latino) (34.1), and Asians (30.0).[2] Racial/ ethnic variation in deaths due to CRC show similar patterns with the highest rates in Black Americans, followed by AI/AN.[2] Northern Plains American Indians (NPAI) men and women are a subgroup of AI/AN and have the highest and second-highest cancer incidence rate among all AI/AN population groups in the US; the group is two times more likely to develop CRC than non-Latino whites in the region.[3,4]

Screening reduces CRC mortality. Screening for CRC reduces both incidence of and mortality from disease by detecting precancerous and cancerous lesions. Approximately 50% of the decline in CRC incidence and mortality between 1975 and 2000 is attributed to screening.[5] Most CRC deaths are preventable through early detection, but suboptimal uptake and completion substantially impair effectiveness. While routine screening for CRC is recommended by the US Preventive Services Task Force (USPSTF) and other medical organizations for individuals age 50 - 75 years (American Cancer Society (ACS) recommends initiating at 45 years), only 67% of Americans are up to date with CRC screening.[6,7]

There are many options for CRC screening. Colonoscopy is the most common CRC screening modality in the US and is considered high value as it provides both diagnostic and therapeutic benefit.[8] Through the resection of pre-cancerous lesions, colonoscopy is associated with a decrease in incidence and mortality of CRC.[9,10] Advantages of colonoscopy include diagnosis and treatment of a lesion during one session and the extended interval between screening. Disadvantages include greater cost, need for full bowel preparation, procedural complications, time necessitated off from work and limited access to an endoscopy center. These access barriers disproportionally affect racial/ethnic and low socioeconomic status (SES) individuals, recent immigrants, and uninsured/Medicaid populations, all of whom have lower colonoscopy rates compared to White, high SES and private /Medicare groups.[1] Particularly in community settings, including federally qualified health centers (FQHCs), access to colonoscopy is a major barrier to colonoscopic screening.[11-13]

The second most common screening test used in the US is the fecal immunochemical tests (FIT).[14] FIT and high sensitivity fecal occult blood testing (FOBT) are annual, non-invasive, at-home tests that detect

hemoglobin in the stool. Among underserved populations who are not up-to-date on CRC screening, outreach methods have led to higher screening completion rates with FIT compared to colonoscopy.[15] Mailed outreach of stool tests versus clinic-based offers for stool tests resulted in a 28% absolute increase in screening completion, with similar results observed in settings serving low income and racial/ethnic minority populations compared to more affluent populations.[16] Particularly during the COVID-19 pandemic, these tests have been critical as they allow for decreased interaction with the healthcare system.[17]

Cologuard© (Exact Sciences), the only FDA approved FIT-DNA stool test, is a newer CRC screening modality that has similar logistical advantages to FIT.[8] This screening option is recommended every three years and combines FIT and multi-targeted stool DNA testing to detect genetic abnormalities in stool. An advantage to this CRC screening modality is that it has the highest one-time sensitivity among non-invasive CRC screening options. The greatest disadvantage is the high cost compared to FIT as it can be up to 10 times more expensive and not all insurance carriers provide coverage. As a result, clinics, especially in resource-limited settings, have not yet incorporated specific workflows to encourage its use. FIT-DNA stool testing also has a lower specificity than FIT alone. However, unlike other screening modalities, Cologuard supports screening completion through their 24/7 patient support system with interpreter services for >200 languages.[8] These extensive patient navigation efforts have resulted in Cologuard completion rates ranging from 88–91% among Medicare beneficiaries.[18,19]

<u>CRC screening rates are lowest among individuals with low levels of education and income, no health insurance, poor access to care, and who are from racial/ethnicity minority backgrounds.</u>[14] AI/AN have the second highest incidence of and mortality from CRC but the lowest screening rates (AI – 62.1%). Screening rates are also lower in Latino (56.1%) and Asian (64.8%) populations than in White populations (71.1%). These disparities are particularly concerning given that Latinos and Asians are the largest and fastest growing populations in the US.[20,21] Although the Black-White screening gap has narrowed in recent years, screening rates among Black Americans (70.0%) remain below the national benchmark of 80%, which is notable given the high incidence and mortality in this group.[14] In 2018, the National Colorectal Cancer Roundtable (NCCRT) announced the “80% in Every Community” campaign, recognizing that in order to realize the full potential of CRC screening to reduce morbidity and mortality in the US, we must address low screening participation in populations with suboptimal rates and the most unfavorable CRC outcomes.[22]

<u>Colonoscopic follow-up after an abnormal stool-based screening test is necessary but suboptimal.</u> Abnormal (i.e. positive) stool-based screening (e.g. FIT, FOBT, Cologuard) results are common (5-14%) and necessitate subsequent diagnostic colonoscopy to assess for pre-cancerous or cancerous lesions.[23] In many circumstances, however, colonoscopic follow-up after an abnormal stool-based test result is delayed or not performed, and there is broad variation in follow-up rates across clinical settings.[24-31] Failure to complete a diagnostic colonoscopy after an abnormal stool-based test is associated with increased CRC incidence, late-stage diagnosis, limited treatment options, and increased CRC-related mortality.[32-35]

<u>Community Health Centers (CHCs)</u> including FQHCs and FQHC-“look alikes”[36] (i.e. community-based centers that meet the requirements of the federal program, but are not funded through this program) provide primary care to a substantial proportion of low-income and uninsured individuals in the US. CHCs serve 1 in 12 people, and are critical to national efforts to address disparities.[37-41] Although CHCs are dedicated to providing evidence-based care, they are often under-resourced. Safety-net populations, like those served in CHCs, have among the lowest rates of CRC screening.[42] In CHCs, like other settings where screening colonoscopy is not feasible or easily accessible, stool-based screening has emerged as a common and inexpensive strategy.[43-46] These populations also have the lowest rates of

diagnostic colonoscopy following an abnormal stool-based screening result (40%-58%), substantially shy of the US Multi-Society Task Force (MSTF) on Colorectal Cancer benchmark to achieve colonoscopy in at least 80% of patients with an abnormal stool-based test.[23,26,30,47-50]

Published data also highlight some of the patient, provider, and system factors associated with lack of colonoscopic follow-up for patients who receive care in CHCs,[26,47-50] including staffing and resource constraints, inadequate referral systems for colonoscopy, and challenges of coordinating care with specialists (gastroenterologists) in outside healthcare systems.[26,47-51] Currently, follow-up colonoscopy is considered a diagnostic procedure (*versus* screening) so many underserved/underinsured patients decline due to cost.[52] While interventions to improve follow-up after abnormal stool-based screening have been described, most of the effective interventions tested in non-CHC settings are unrealistic for CHC due to resource limitations.[53,54] To improve follow-up rates, there is a need for research that systematically addresses challenges in the processes of care required for successful follow-up in CHC settings.

Effective <u>patient</u>-directed interventions have included direct patient outreach (e.g., telephone, mailings), education through small-media printed materials (e.g., brochures, newsletters), decision aids, and reminders (e.g., telephone, mailed, electronic).[56] Mailed CRC screening outreach, in particular, has been very effective in CHC settings and has been promoted during the COVID-19 pandemic.[17,57] Patient interventions have also included patient navigation of the screening process, and barrier-directed efforts.[58,59] <u>Provider</u>-directed interventions are less common but also effective. At the provider-level, interventions have used education (e.g., training modules, workshops), financial incentives, electronic health record (EHR) alerts/reminders to encourage providers to recommend CRC screening, all with variable success.[59,60] Interventions that are aimed to optimize <u>health systems</u> for CRC screening include adaptations to the EHR to optimize documentation of screening recommendations and measurement of screening rates.[56,58,59,61] System-level interventions also include improvements to clinic workflow and incorporation of system-wide screening dashboards and quality improvement programs to increase screening rates.[56,58,59]

Patient Navigation (PN) is an evidence-based health system approach for coordinating care, designed to ensure that necessary care is delivered to all patients including the most vulnerable.[62-68] Services offered by PN programs are flexible and focus on individual problem-solving rather than providing a pre-defined set of services. PN has been shown to improve rates of cancer screening, particularly for safety-net populations.[65,69,70] PN is traditionally delivered by a combination of phone calls and face-to-face visits,[64,71] often making broad screening navigation programs difficult for individual clinical sites to maintain.[53,54,71,72] Shared or centralized navigation across CHCs may be more sustainable when focused on ensuring timely diagnostic evaluation.

Overall, the most successful interventions to improve screening and diagnostic follow-up are multi-component and multi-level interventions that address patient, provider, and health system barriers to CRC screening.[73,74] Furthermore, of the evidence-based interventions evaluated in low-income and racial/ ethnic minority populations, those that include mailed outreach with stool-based screening kits and screening reminders, navigation, or patient support have been most effective.[61,73,75]

<u>The COVID-19 pandemic has substantially increased the challenges of providing timely CRC screening</u>, particularly in the populations served by CHCs.[17,76-78] These populations have been disproportionately affected by COVID-19 related illness, job loss and other economic sequelae, and may be more hesitant to accept the COVID-19 vaccine.[79,80] The COVID pandemic has only served to markedly increase the barriers to colonoscopy as access has been severely limited resulting in a backlog of patients waiting for procedures, particularly for patients served by CHCs. Nonetheless, the pandemic has also created an

opportunity to shift the locus of preventive care from in-person visits to a strategy that focuses on population health with more widespread deployment of home-based testing with robust community-based strategies involving navigators to overcome barriers to care for underserved populations.[81]

2. Specific Aims and Objectives

Aim 1: Develop and conduct a 2-arm, multi-level, multi-component pragmatic trial randomized at the level of the community health center (CHC) to compare two population outreach approaches to increase CRC screening uptake among screening eligible adults: (1) Mailed Cologuard, with support program, and (2) Mailed FIT outreach with systematic reminders. Mailed Cologuard with support program is the standard of care for patients prescribed Cologuard in any setting and includes telephone, text, and email support. Mailed FIT with systematic reminders is an evidence-based approach that will supplement population outreach through additional sustainable, approaches.

The study will be conducted in 8,000 individuals across 10 CHCs in greater-Boston, Los Angeles County (LA), and Tribal Nations in South Dakota (SD). **This IRB application pertains to estimated 3200 individuals from 4 CHCs that are part of the MGH system.**

Primary Outcome: The completion rate of the stool-based screening test (Cologuard or FIT) at 3 months for each study arm.

Secondary Outcomes: Completion rate of the stool-based screening test will also be assessed at 6 months for each study arm. Implementation outcomes including reach of each screening modality among eligible patients, feasibility and acceptability based on feedback from patients, providers, and staff.

Hypotheses include: (1) CRC screening completion rates will increase in CHCs in both study arms; (2) Cologuard will have a higher completion rate than FIT because of the extensive outreach and the appeal of the 3-year screening interval; and (3) population-based outreach with both stool-based strategies will be feasible and acceptable in diverse CHC settings.

Aim 2a) Increase completion of diagnostic evaluation after abnormal Cologuard or FIT by offering a virtual patient navigation program (phone or video) to address barriers to follow-up among all individuals in Aim 1 with an abnormal result.

Primary outcome: The rate of completion of diagnostic colonoscopy, overall and by arm, within 6 months of an abnormal stool test result.

Hypotheses include: (1) Diagnostic colonoscopy completion will increase compared to historical rates and will be similar in both study arms; (2) patient navigation for diagnostic evaluation will be feasible and acceptable in CHCs.

3. General Description of Study Design

The CHC pragmatic intervention has four levels, including system-, provider-, patient-, and community-level components at each of the CARES clinical sites. **Health system components** will include the development and implementation of a CRC screening registry and tracking database to systematically

and actively track patients overdue for screening, screening test completion, and abnormal screening results. Data systems will be coupled with process re-design to improve clinical workflow for screening outreach. **Providers** will receive educational support through a training session about the importance of CRC screening, screening test options, population management, and an instructional on how to complete a Cologuard kit (Cologuard arm only) or a FIT kit (FIT arm only). **Patient components** of the intervention vary by study arm. In sites randomized to Cologuard, patients will receive a mailed Cologuard kit and the Exact Sciences patient support program. Patients in clinics randomized to FIT will receive a mailed FIT kit with systematic reminders.

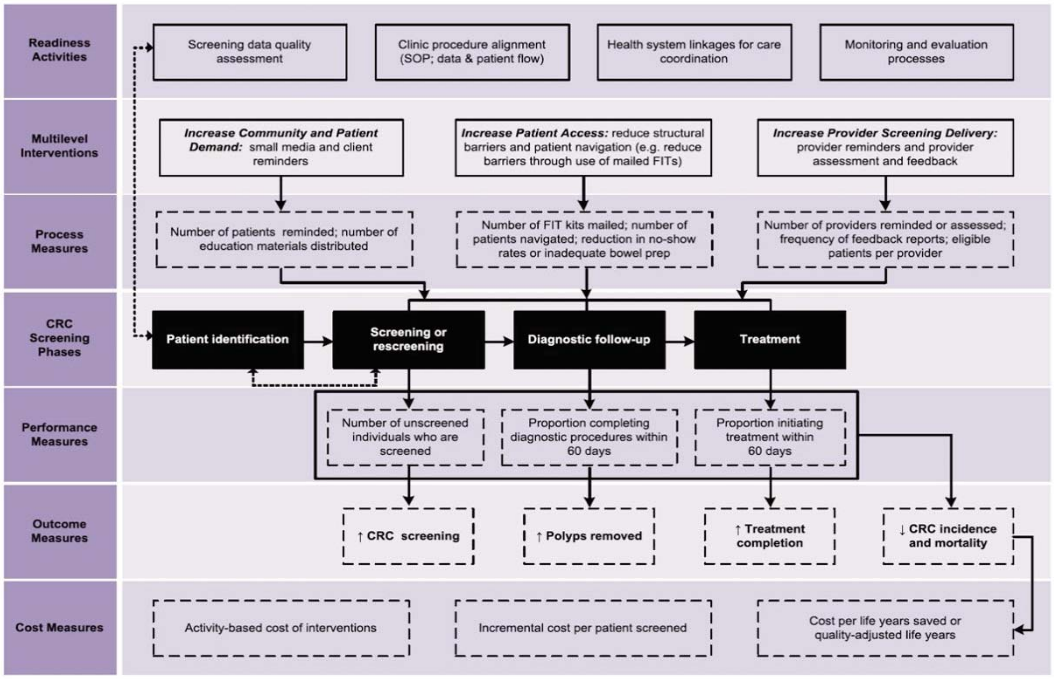

Figure 1. This is the conceptual framework for implementing and evaluating multicomponent colorectal cancer (CRC) interventions. FIT indicates fecal immunochemical test; SOP, standard operating procedure.

4. Subject Selection

Trial: Individuals in the designated CHCs will be eligible if they are between the ages of 45 to 75 years and are overdue for CRC screening (have not received FIT or FOBT test in past year, Cologuard in the prior 3 years, or colonoscopy in past 10 years). We will exclude individuals who have a prior diagnosis of CRC or individuals who are under surveillance for a prior abnormal colonoscopy result (i.e., polyps).

Eligible individuals will come from one of four CHCs affiliated with Mass General Brigham and sharing a common EHR in the greater-Boston area (1) _Chelsea_ (2) _Charlestown,_ (3) _Brookside_ and (4) _Southern Jamaica Plain_. These CHC's disproportionately care for minority and low-income populations that were particularly hard hit by the pandemic. As of November 2020, less than 50% of screening eligible patients were up-to-date with CRC screening at the participating health centers. These four Boston sites have approximately 12,500 screening eligible patients. Of the individuals due for CRC screening, 60.7% are white, 25.2% Latino, 10.3% Black and 3.8% Asian.

5. Subject Enrollment

AIM 1. Develop and conduct a 2-arm, multi-level, multi-component pragmatic trial randomized at the level of the community health center (CHC) to compare two CRC population screening outreach approaches for screening eligible adults.

System level: (1) We have developed common definitions and protocols to identify and track eligible patients at each CHC using EHR/ data systems; (2) Worked with CHC around process re-design to develop a population management approach that balances CHC workflow and staffing and ensures study protocol fidelity (see protocol #: 2021P002491, 2022P000206). A similar process will be conducted for both study arms, tailored to the randomization status.

Provider/staff level: In both arms, the study team will work with participating clinics to ensure providers and staff have the knowledge and skills necessary to maintain a robust CRC screening program after the project is completed. We will focus on sharing best practices to improve CRC screening and diagnostic evaluation, including establishing and maintaining a population management system to support CRC screening.

Patient-level: For patients who receive care in a clinic randomized to FIT, the CARES Team has developed brief motivational text messages that will be used to remind patients to return the FIT kit. These text messages are modelled after those used for other successful stool-based CRC outreach in English and Spanish.[121] All patients will remain under the care of their primary care team and the intervention components will be delivered in addition to those individuals who are overdue for follow-up.

Aim 2a: We anticipate that approximately 8% of people who participate in Aim 1 will have an abnormal stool-based screening result,[23] resulting in approximately 320 individuals at MGB who will need diagnostic colonoscopy. A recent systematic review of interventions to improve abnormal FIT follow-up concluded there is evidence of moderate strength for patient navigation and provider reminders as strategies for increasing colonoscopy completion.[132]

6. STUDY PROCEDURES

Randomization Scheme. We will perform a 2-arm pragmatic trial with randomization at the CHC-level. We have chosen this level of randomization as it would be logistically complicated for the practices to have some of their patients randomized to receive Cologuard and some of their patients randomized to receive FIT.

Intervention Delivery. To be efficient for clinic workflow, eligible patients will be identified in waves over six months. For each wave, study staff will identify eligible individuals by querying their EHR or population management system. Staff will then perform an arm-specific study protocol for each eligible patient. Patients will be sent information through the mail. The mailing will include a letter from the medical director of each CHC, a one-page information sheet on the importance of colorectal cancer screening related to FIT or Cologuard depending on the study arm the CHC was assigned to, and an opt-out letter that describes the project signed by the PI. Patients will be asked to opt-out within two weeks by contacting the study staff by phone and or email.

Cologuard Arm: In these clinics, a list of patients (with patient contact information and name of primary care provider) identified as eligible for screening by the CRC screening registry will be sent to Exact Sciences using a secure portal called EpicCare Link (MGB cybersecurity review approved) so that each eligible patient can receive a mailed Cologuard Kit with a pre-addressed and postage-paid container in which to return the kit to the Exact Sciences laboratory. These patients will be enrolled into the 4-week Exact Sciences Patient Compliance Program (standard of care, delivered in clinical settings), developed and implemented by Exact Sciences to maximize Cologuard kit completion rates.[131] Once a kit is returned to Exact Sciences and processed, the results will be available in the EpicCare Link portal. Study staff will use a unique username and password to access the results. All results will be exported as pdfs from the EpicCare Link system and emailed to PHSHIMScanning@partners.org in the MGB Digital Health Information Management group. A process has been developed to create a new order, import the file and modify the result (see attached in Other as Cologuard Workflow for HIMS).

FIT arm: Patients identified as eligible for screening and who have not opted out will receive a mailed FIT kit with a pre-addressed, postage-paid return envelope so that the completed FIT is returned to the clinic lab for processing. The mailing will also include a letter from their primary care provider at the CHC. Instructions for FIT completion will be simple and address common problems that may lead to failed laboratory processing, such as lack of notation of a collection date and kit overfilling.[132] The kit will include a 1-page information sheet with graphical instructions for how to perform the test. Patients will be encouraged to return the kit within 2 weeks. Because these clinics do not have the resources/ staffing to do broad phone outreach to patients, we will use texting as our primary outreach modality.

“Primers” like a text before receiving a FIT kit increase overall adherence.[132] Text message content will be based on tested messages to reach the under-screened, developed by the NCCRT for their “80% in Every Community” campaign and available in English and Spanish.[133] We will use the approved HIPAA-compliant email-to-text process . A text message is sent via MGB email to the patient’s listed cell phone. This process requires a cell phone carrier verification step (Via Twilio) before the message is sent, and messages will be sent in small batches.

Patients will receive up to 3 text messages to remind them to return the FIT kits. The following is the process we will use to text patients. Once we retrieve a phone number from Epic, we will verify the phone number carrier (for example, ATT, Verizon, etc) using a Twilio account. There is a look up table in Twilio. The carrier is used to determine the extension for the email address. We will create an email address that includes the phone number and the carrier. We then send an email using the address we created by combining the phone number and carrier extension. If someone opts out, the message will go back to the email account that sent the message and we will manage those replies. Patients will be sent a text prior to receiving the fit kit and then two and four weeks after the fit kit has been mailed.

A process has been created with the DHeC Research Team to upload the list of eligible patients identified from Epic workbenches and assign them to an Epic Research Study using an enrollment template. Study staff will open a help desk ticket and assign it to the DHeC Research Team, who will upload the list into Epic and automatically enroll the patients to the Epic Research Study. This is a necessary step so that all the patients in this study can be assigned to Dr. Jennifer Haas as the Authorizing Provider for processing the FIT test and that a primary care provider co-signature will not be needed. All resulted orders will be sent to Dr. Jennifer Haas and the patient’s primary care provider. This process will also allow an Open Order to be generated, a necessary step for the FIT results to be processed. Having the patients in an Epic study register will also allow study staff to run a Research Billing Review Report to monitor the billing status of any patients who do not have insurance.

Data Sources. *Electronic Health Records (EHR)* at each of the CHCs will be used to identify eligible patients. We have worked with the ECare team to develop CHC-specific workbenches to identify eligible patients and track completion of CRC screening. The EHR will also be our primary source of outcome data for both arms, including: screening data (date of completion if any, screening modality used), demographic characteristics (age, sex, race, ethnicity, education, primary language, insurance coverage) clinical characteristics (, prior history of cancer, family history of CRC), and prior CRC screening if any (date, modality).

All patients will be sent an initial letter that will present the elements of consent. The letter will instruct patients how to 'opt-out' of the study if they do not want to participate. All patients will remain under the care of their primary care team and patients can chose any CRC screening modality in discussion with their health care team regardless of which arm their clinic is randomized to.

2a) Navigation for patients with abnormal test results: The CARES team has worked with each CHC to create a process to identify and track patients in need of diagnostic evaluation, ensure that these individuals are informed of their results and referred to colonoscopy, and train CHC staff to track colonoscopy completion status. All patients will receive colonoscopy navigation. The navigator will assist with insurance enrollment if needed, facilitate care coordination with the primary care team, gastroenterologists, surgeons and/or oncologists that partner with the CHCs, document whether a colonoscopy has been received, and document colonoscopy findings (e.g., advanced adenomas and cancers). Navigators will document persistent barriers to colonoscopic follow-up and oncologic care to inform future work in this understudied and highly important research area.

Data Collection: We will establish a robust, secure, web-based dashboard programmed in Access, to manage data collected at each site. The Dashboard will be used by the navigators to track outreach, persistent barriers, and completion of colonoscopy. As part of the navigation process, patients will be asked several questions by the patient navigator to calculate the patient's risk of cancer (including personal and family history of cancer). The tool being used is the PREMM scale. The risk calculator determines the patient's risk as low, moderate or high and patients will receive a document describing their level of risk. The PREMM questions and the risk assessment output are included as Other attachments.

For training purposes and to monitor the quality of the patient navigator phone calls, we will record the first 20 cases navigated and up to a randomly selected 15% of the navigator calls, which will be reviewed by Dr. Jennifer Haas.

For patients who complete a diagnostic colonoscopy following a positive stool test, we will review their endoscopy and pathology reports from Epic. We will extract the data about the colonoscopy findings. This information will be used to describe the findings detected in the two arms of the study.

Remuneration:

Subjects in the main trial will not receive remuneration.

Translation:

Once study materials are finalized, all patient-facing materials will be translated into Spanish by an IRB approved vendor and submitted for final approval before they are used.

7. Risks and Discomforts

We believe that all of the risks described are uncommon. Potential risks to subjects include loss of confidentiality of healthcare data. Study staff will follow careful protocols to minimize these risks. The co-investigators will emphasize the importance of maintaining confidentiality in the training of all study staff. All study data and survey questionnaires will be coded with unique study identification numbers. Electronic data will be stored within the MGB firewalls, will be password protected, and will be protected by anti-virus software. Only study staff will have access to study data on shared file areas.

While unlikely, some patients may be contacted during the trial based upon inaccurate or incomplete information in her/his electronic health record. There may be psychological stress associated with such contact, but information provided by patients will be used to update the patient's record/ notify the patient's care team, thus ultimately resulting in better quality care. Study staff will specifically be trained to help patients cope with these issues. Study staff, particularly the navigators, will be trained to specifically address any personal stressors that a patient may have that is interfering with their ability to get needed care. During the trial phase, patients are only indirectly affected through process of care modifications at the practices they attend – they can continue to get any CRC screening modality that they prefer in consultation with their providers beyond the population based outreach being tested in this protocol. We do not anticipate physical risks to patients as a result of participation.

8. Benefits

Participants in practices randomly assigned to either intervention arm may benefit by receiving more timely screening and follow-up of their abnormal CRC screening test result. If the intervention is effective, more timely screening and follow-up of abnormal CRC screening test results could lead to earlier detection, treatment, and cure of CRC. In the future, all patients could benefit from the knowledge produced by this study through the dissemination of similar care systems.

9. Statistical Analysis

Our comparative analyses between the intervention arms will employ the intent-to-treat principle. With the cluster randomized design, we will compare the proportions of eligible individuals who complete screening between intervention arms using a logistic regression model with the generalized estimating equations (GEE) approach to account for the correlated nature of the data within clinics. Variables found to differ between the intervention arms will be further evaluated to assess their confounding effects on

outcomes and included in the logistic regression model if necessary. Two approaches will be considered for adjusting for the intra-cluster correlation: (1) use a robust sandwich covariance matrix estimate to account for the intra-cluster dependence[140] and (2) use a shared frailty model where cluster effects are incorporated into the model as independent and identically distributed random variables.[141]

10. Monitoring and Quality Assurance

The PI will be responsible for monitoring the safety and effectiveness of this trial and complying with the reporting requirements.

Data monitoring plan
All data collection/ storage systems will be piloted before the study begins. Any patient data collected as part of the study itself will be stored electronically behind the MGB firewall with password protection and anti-virus software. Only study staff will have access to the study data on Shared File Areas. The PIs will be responsible for monitoring and assuring the validity and integrity of the data and adherence to the IRB-approved protocol.

Safety monitoring plan
The main safety risks for the study include the potential for psychological discomfort associated with the intervention. Though this should not differ by study arm. The benefits of receiving timely CRC screening follow-up of an abnormal screening tests substantially outweigh this risk. We will monitor complaints received from patients and/or providers and notify the IRB as required by local governance. The main risk of the study is unintended release of patient health information collected and maintained by the study investigators. As noted, we will apply rigorous data safety and monitoring standards to ensure that this does not occur.

11. Privacy and Confidentiality

☒ Study procedures will be conducted in a private setting
☒ Only data and/or specimens necessary for the conduct of the study will be collected
☒ Data collected (paper and/or electronic) will be maintained in a secure location with appropriate protections such as password protection, encryption, physical security measures (locked files/areas)
☒ Specimens collected will be maintained in a secure location with appropriate protections (e.g. locked storage spaces, laboratory areas)
☒ Data and specimens will only be shared with individuals who are members of the IRB-approved research team or approved for sharing as described in this IRB protocol
☒ Data and/or specimens requiring transportation from one location or electronic space to another will be transported only in a secure manner (e.g. encrypted files, password protection, using chain-of-custody procedures, etc.)
☒ All electronic communication with participants will comply with Mass General Brigham secure communication policies

☒ Identifiers will be coded or removed as soon as feasible and access to files linking identifiers with coded data or specimens will be limited to the minimal necessary members of the research team required to conduct the research
☒ All staff are trained on and will follow the Mass General Brigham policies and procedures for maintaining appropriate confidentiality of research data and specimens
☒ The PI will ensure that all staff implement and follow any Research Information Service Office (RISO) requirements for this research
☒ Additional privacy and/or confidentiality protections

To ensure patient privacy and confidentiality, all information will be stored within the MGB firewall, password protected, and anti-virus software enabled. Only study staff will have access to the study data on Shared File Areas. Only de-identified data will be used for the purposes of publication or presentation.

12. References

1. Siegel RL, Miller KD, Jemal A. Cancer statistics, 2020. *CA Cancer J Clin.* 2020;70(1):7-30.
2. Siegel RL, Miller KD, Goding Sauer A, et al. Colorectal cancer statistics, 2020. *CA: a cancer journal for clinicians.* 2020.
3. White MC, Espey DK, Swan J, Wiggins CL, Eheman C, Kaur JS. Disparities in cancer mortality and incidence among American Indians and Alaska Natives in the United States. *Am J Public Health.* 2014;104 Suppl 3:S377-387.
4. Wiggins CL, Espey DK, Wingo PA, et al. Cancer among American Indians and Alaska Natives in the United States, 1999-2004. *Cancer.* 2008;113(5 Suppl):1142-1152.
5. Zauber AG. The impact of screening on colorectal cancer mortality and incidence: has it really made a difference? *Dig Dis Sci.* 2015;60(3):681-691.
6. Wolf AMD, Fontham ETH, Church TR, et al. Colorectal cancer screening for average-risk adults: 2018 guideline update from the American Cancer Society. *CA: a cancer journal for clinicians.* 2018;68(4):250-281.
7. Bibbins-Domingo K, Grossman DC, Curry SJ, Davidson KW, Epling JW, Jr., Garcia FAR, et al. Screening for Colorectal Cancer: US Preventive Services Task Force Recommendation Statement. Jama. 2016;315(23):2564-75.
8. Rex DK, Boland CR, Dominitz JA, et al. Colorectal Cancer Screening: Recommendations for Physicians and Patients from the U.S. Multi-Society Task Force on Colorectal Cancer. *Am J Gastroenterol.* 2017;112(7):1016-1030.
9. Nishihara R, Wu K, Lochhead P, et al. Long-term colorectal-cancer incidence and mortality after lower endoscopy. *N Engl J Med.* 2013;369(12):1095-1105.
10. Winawer SJ, Zauber AG, Ho MN, et al. Prevention of colorectal cancer by colonoscopic polypectomy. The National Polyp Study Workgroup. *N Engl J Med.* 1993;329(27):1977-1981.
11. Inadomi JM, Vijan S, Janz NK, et al. Adherence to colorectal cancer screening: a randomized clinical trial of competing strategies. *Arch Intern Med.* 2012;172(7):575-582.
12. Montminy EM, Karlitz JJ, Landreneau SW. Progress of colorectal cancer screening in United States: Past achievements and future challenges. *Prev Med.* 2019;120:78-84.

13. Towne SD, Jr., Smith ML, Ory MG. Geographic variations in access and utilization of cancer screening services: examining disparities among American Indian and Alaska Native Elders. *Int J Health Geogr.* 2014;13:18.
14. Joseph DA, King JB, Dowling NF, Thomas CC, Richardson LC. Vital Signs: Colorectal Cancer Screening Test Use - United States, 2018. *MMWR Morb Mortal Wkly Rep.* 2020;69(10):253-259.
15. Gupta S, Halm EA, Rockey DC, et al. Comparative effectiveness of fecal immunochemical test outreach, colonoscopy outreach, and usual care for boosting colorectal cancer screening among the underserved: a randomized clinical trial. *JAMA Intern Med.* 2013;173(18):1725-1732.
16. Jager M, Demb J, Asghar A, et al. Mailed Outreach Is Superior to Usual Care Alone for Colorectal Cancer Screening in the USA: A Systematic Review and Meta-analysis. *Dig Dis Sci.* 2019;64(9):2489-2496.
17. Balzora S, Issaka RB, Anyane-Yeboa A, Gray DM, 2nd, May FP. Impact of COVID-19 on colorectal cancer disparities and the way forward. *Gastrointest Endosc.* 2020;92(4):946-950.
18. Weiser E, Parks PD, Swartz RK, et al. Cross-sectional adherence with the multi-target stool DNA test for colorectal cancer screening: Real-world data from a large cohort of older adults. *J Med Screen.* 2020:969141320903756.
19. Prince M, Lester L, Chiniwala R, Berger B. Multitarget stool DNA tests increases colorectal cancer screening among previously noncompliant Medicare patients. *World J Gastroenterol.* 2017;23(3):464-471.
20. U.S. Census Bureau. Population Projections. 2014 National Population Projections: Summary Tables. https://www.census.gov/population/projections/data/national/2014/summarytables.html. Accessed.
21. U.S. Census Bureau. State and County QuickFacts. Data derived from Population Estimates, American Community Survey, Census of Population and Housing, State and County Housing Unit Estimates, County Business Patterns, Nonemployer Statistics, Economic Census, Survey of Business Owners, Building Permits. 2015 February 05. http://www.census.gov/quickfacts Accessed.
22. Wender R, Brooks D, Sharpe K, Doroshenk M. The National Colorectal Cancer Roundtable: Past Performance, Current and Future Goals. *Gastrointest Endosc Clin N Am.* 2020;30(3):499-509.
23. Robertson DJ, Lee JK, Boland CR, et al. Recommendations on Fecal Immunochemical Testing to Screen for Colorectal Neoplasia: A Consensus Statement by the US Multi-Society Task Force on Colorectal Cancer. *The American journal of gastroenterology.* 2017;112(1):37-53.
24. Gellad ZF, Almirall D, Provenzale D, Fisher DA. Time from positive screening fecal occult blood test to colonoscopy and risk of neoplasia. *Dig Dis Sci.* 2009;54(11):2497-2502.
25. Partin MR, Burgess DJ, Burgess JF, Jr., et al. Organizational predictors of colonoscopy follow-up for positive fecal occult blood test results: an observational study. *Cancer Epidemiol Biomarkers Prev.* 2015;24(2):422-434.
26. Issaka RB, Singh MH, Oshima SM, et al. Inadequate Utilization of Diagnostic Colonoscopy Following Abnormal FIT Results in an Integrated Safety-Net System. *The American journal of gastroenterology.* 2017;112(2):375-382.
27. Powell AA, Gravely AA, Ordin DL, Schlosser JE, Partin MR. Timely follow-up of positive fecal occult blood tests strategies associated with improvement. *Am J Prev Med.* 2009;37(2):87-93.
28. Correia A, Rabeneck L, Baxter NN, et al. Lack of follow-up colonoscopy after positive FOBT in an organized colorectal cancer screening program is associated with modifiable health care practices. *Prev Med.* 2015;76:115-122.

29. Tosteson AN, Beaber EF, Tiro J, et al. Variation in Screening Abnormality Rates and Follow-Up of Breast, Cervical and Colorectal Cancer Screening within the PROSPR Consortium. *J Gen Intern Med.* 2016;31(4):372-379.
30. Bharti B, May FFP, Nodora J, et al. Diagnostic colonoscopy completion after abnormal fecal immunochemical testing and quality of tests used at 8 Federally Qualified Health Centers in Southern California: Opportunities for improving screening outcomes. *Cancer.* 2019.
31. Chubak J, Garcia MP, Burnett-Hartman AN, et al. Time to Colonoscopy after Positive Fecal Blood Test in Four U.S. Health Care Systems. *Cancer Epidemiol Biomarkers Prev.* 2016;25(2):344-350.
32. Forbes N, Hilsden RJ, Martel M, et al. Association Between Time to Colonoscopy After Positive Fecal Testing and Colorectal Cancer Outcomes: A Systematic Review. *Clin Gastroenterol Hepatol.* 2020.
33. San Miguel Y, Demb J, Martinez ME, Gupta S, May FP. Time to Colonoscopy After Abnormal Stool-Based Screening and Risk for Colorectal Cancer Incidence and Mortality. *Gastroenterology.* 2021.
34. Corley DA, Jensen CD, Quinn VP, et al. Association Between Time to Colonoscopy After a Positive Fecal Test Result and Risk of Colorectal Cancer and Cancer Stage at Diagnosis. *JAMA.* 2017;317(16):1631-1641.
35. Robertson DJ, Lee JK, Boland CR, et al. Recommendations on Fecal Immunochemical Testing to Screen for Colorectal Neoplasia: A Consensus Statement by the US Multi-Society Task Force on Colorectal Cancer. *Gastroenterology.* 2017;152(5):1217-1237 e1213.
36. Federally Qualified Health Center Look-Alike. https://www.hrsa.gov/opa/eligibility-and-registration/health-centers/fqhc-look-alikes/index.html. Accessed February 2, 2021.
37. Centers for Disease Control and Prevention (CDC). Behavioral Risk Factor Surveillance System Survey Questionnaire. Atlanta GUSDoHaHS, Centers for Disease Control and Prevention, 2014.
38. Doubeni CA, Laiyemo AO, Major JM, et al. Socioeconomic status and the risk of colorectal cancer: an analysis of more than a half million adults in the National Institutes of Health-AARP Diet and Health Study. *Cancer.* 2012;118(14):3636-3644.
39. Doubeni CA, Major JM, Laiyemo AO, et al. Contribution of behavioral risk factors and obesity to socioeconomic differences in colorectal cancer incidence. *Journal of the National Cancer Institute.* 2012;104(18):1353-1362.
40. Manser CN, Bauerfeind P. Impact of socioeconomic status on incidence, mortality, and survival of colorectal cancer patients: a systematic review. *Gastrointest Endosc.* 2014;80(1):42-60 e49.
41. Tammana VS, Laiyemo AO. Colorectal cancer disparities: issues, controversies and solutions. *World journal of gastroenterology : WJG.* 2014;20(4):869-876.
42. Gupta S, Tong L, Allison JE, et al. Screening for colorectal cancer in a safety-net health care system: access to care is critical and has implications for screening policy. *Cancer Epidemiol Biomarkers Prev.* 2009;18(9):2373-2379.
43. Lee JK, Liles EG, Bent S, Levin TR, Corley DA. Accuracy of fecal immunochemical tests for colorectal cancer: systematic review and meta-analysis. *Ann Intern Med.* 2014;160(3):171.
44. Zorzi M, Fedeli U, Schievano E, et al. Impact on colorectal cancer mortality of screening programmes based on the faecal immunochemical test. *Gut.* 2015;64(5):784-790.
45. Chiu HM, Chen SL, Yen AM, et al. Effectiveness of fecal immunochemical testing in reducing colorectal cancer mortality from the One Million Taiwanese Screening Program. *Cancer.* 2015;121(18):3221-3229.
46. Chiang TH, Chuang SL, Chen SL, et al. Difference in performance of fecal immunochemical tests with the same hemoglobin cutoff concentration in a nationwide colorectal cancer screening program. *Gastroenterology.* 2014;147(6):1317-1326.

47. Thamarasseril S, Bhuket T, Chan C, Liu B, Wong RJ. The Need for an Integrated Patient Navigation Pathway to Improve Access to Colonoscopy After Positive Fecal Immunochemical Testing: A Safety-Net Hospital Experience. *J Community Health.* 2017;42(3):551-557.
48. Martin J, Halm EA, Tiro JA, et al. Reasons for Lack of Diagnostic Colonoscopy After Positive Result on Fecal Immunochemical Test in a Safety-Net Health System. *Am J Med.* 2017;130(1):93 e91-93 e97.
49. McCarthy AM, Kim JJ, Beaber EF, et al. Follow-Up of Abnormal Breast and Colorectal Cancer Screening by Race/Ethnicity. *Am J Prev Med.* 2016;51(4):507-512.
50. Oluloro A, Petrik AF, Turner A, et al. Timeliness of Colonoscopy After Abnormal Fecal Test Results in a Safety Net Practice. *J Community Health.* 2016;41(4):864-870.
51. Sarfaty M, Doroshenk M, Hotz J, et al. Strategies for expanding colorectal cancer screening at community health centers. *CA: a cancer journal for clinicians.* 2013;63(4):221-231.
52. Percac-Lima S. Capsule Commentary on Jetelina et al., Patient-Reported Barriers to Completing a Diagnostic Colonoscopy Following Abnormal Fecal Immunochemical Test among Uninsured Patients. *J Gen Intern Med.* 2019;34(9):1851.
53. Raich PC, Whitley EM, Thorland W, Valverde P, Fairclough D, Denver Patient Navigation Research P. Patient navigation improves cancer diagnostic resolution: an individually randomized clinical trial in an underserved population. *Cancer epidemiology, biomarkers & prevention : a publication of the American Association for Cancer Research, cosponsored by the American Society of Preventive Oncology.* 2012;21(10):1629-1638.
54. Selby K, Baumgartner C, Levin TR, et al. Interventions to Improve Follow-up of Positive Results on Fecal Blood Tests: A Systematic Review. *Ann Intern Med.* 2017;167(8):565-575.
55. Subramanian S, Hoover S, Tangka FKL, et al. A conceptual framework and metrics for evaluating multicomponent interventions to increase colorectal cancer screening within an organized screening program. *Cancer.* 2018;124(21):4154-4162.
56. Carethers JM, Doubeni CA. Causes of Socioeconomic Disparities in Colorectal Cancer and Intervention Framework and Strategies. *Gastroenterology.* 2020;158(2):354-367.
57. Coronado GD, Petrik AF, Vollmer WM, et al. Effectiveness of a Mailed Colorectal Cancer Screening Outreach Program in Community Health Clinics: The STOP CRC Cluster Randomized Clinical Trial. *JAMA Intern Med.* 2018;178(9):1174-1181.
58. Tiro JA, Kamineni A, Levin TR, et al. The colorectal cancer screening process in community settings: a conceptual model for the population-based research optimizing screening through personalized regimens consortium. *Cancer epidemiology, biomarkers & prevention : a publication of the American Association for Cancer Research, cosponsored by the American Society of Preventive Oncology.* 2014;23(7):1147-1158.
59. Dougherty MK, Brenner AT, Crockett SD, et al. Evaluation of Interventions Intended to Increase Colorectal Cancer Screening Rates in the United States: A Systematic Review and Meta-analysis. *JAMA Intern Med.* 2018;178(12):1645-1658.
60. Peterson EB, Ostroff JS, DuHamel KN, et al. Impact of provider-patient communication on cancer screening adherence: A systematic review. *Prev Med.* 2016;93:96-105.
61. Issaka RB, Avila P, Whitaker E, Bent S, Somsouk M. Population health interventions to improve colorectal cancer screening by fecal immunochemical tests: A systematic review. *Prev Med.* 2019;118:113-121.
62. Clark CR, Baril N, Kunicki M, et al. Addressing social determinants of health to improve access to early breast cancer detection: results of the Boston REACH 2010 Breast and Cervical Cancer Coalition Women's Health Demonstration Project. *Journal of women's health.* 2009;18(5):677-690.

63. Ramachandran A, Freund KM, Bak SM, Heeren TC, Chen CA, Battaglia TA. Multiple barriers delay care among women with abnormal cancer screening despite patient navigation. *Journal of women's health.* 2015;24(1):30-36.
64. Percac-Lima S, Ashburner JM, McCarthy AM, Piawah S, Atlas SJ. Patient navigation to improve follow-up of abnormal mammograms among disadvantaged women. *Journal of women's health.* 2015;24(2):138-143.
65. Marshall JK, Mbah OM, Ford JG, et al. Effect of Patient Navigation on Breast Cancer Screening Among African American Medicare Beneficiaries: A Randomized Controlled Trial. *Journal of general internal medicine.* 2015.
66. Freund KM. Patient navigation: the promise to reduce health disparities. *Journal of general internal medicine.* 2011;26(2):110-112.
67. Battaglia TA, Burhansstipanov L, Murrell SS, Dwyer AJ, Caron SE. Assessing the impact of patient navigation: prevention and early detection metrics. *Cancer.* 2011;117(15 Suppl):3553-3564.
68. Freeman HP, Rodriguez RL. History and principles of patient navigation. *Cancer.* 2011;117(15 Suppl):3539-3542.
69. Percac-Lima S, Lopez L, Ashburner JM, Green AR, Atlas SJ. The longitudinal impact of patient navigation on equity in colorectal cancer screening in a large primary care network. *Cancer.* 2014;120(13):2025-2031.
70. Roland KB, Milliken EL, Rohan EA, et al. Use of Community Health Workers and Patient Navigators to Improve Cancer Outcomes Among Patients Served by Federally Qualified Health Centers: A Systematic Literature Review. *Health Equity.* 2017;1(1):61-76.
71. Ramachandran A, Freund KM, Bak SM, Heeren TC, Chen CA, Battaglia TA. Multiple barriers delay care among women with abnormal cancer screening despite patient navigation. *J Womens Health (Larchmt).* 2015;24(1):30-36.
72. Behforouz HL. Bridging the gap: a community health program saved lives, then closed its doors. *Health affairs.* 2014;33(11):2064-2067.
73. Force CPST. *Cancer Screening: Multicomponent Interventions—Colorectal Cancer.* 2016.
74. Taplin SH, Anhang Price R, Edwards HM, et al. Introduction: Understanding and influencing multilevel factors across the cancer care continuum. *J Natl Cancer Inst Monogr.* 2012;2012(44):2-10.
75. Davis MM, Freeman M, Shannon J, et al. A systematic review of clinic and community intervention to increase fecal testing for colorectal cancer in rural and low-income populations in the United States - How, what and when? *BMC Cancer.* 2018;18(1):40.
76. Kaufman HW, Chen Z, Niles J, Fesko Y. Changes in the Number of US Patients With Newly Identified Cancer Before and During the Coronavirus Disease 2019 (COVID-19) Pandemic. *JAMA network open.* 2020;3(8):e2017267-e2017267.
77. Mast C, Munoz del Rio A. Delayed Cancer Screenings—A Second Look. https://ehrn.org/articles/delayed-cancer-screenings-a-second-look/. Published 2020. Accessed December 10, 2020.
78. Corley DA, Sedki M, Ritzwoller DP, et al. Cancer Screening during COVID-19: A Perspective from NCI's PROSPR consortium. *Gastroenterology (New York, NY 1943).* 2020.
79. Kreps S, Prasad S, Brownstein JS, et al. Factors Associated With US Adults' Likelihood of Accepting COVID-19 Vaccination. *JAMA Network Open.* 2020;3(10):e2025594-e2025594.
80. COVIDCollaborative, Coronavirus Vaccine Hesitancy in Black and Latinx Communities. In:2020.
81. Horn DM, Haas JS. Covid-19 and the Mandate to Redefine Preventive Care. *N Engl J Med.* 2020;383(16):1505-1507.
