# Supplementary material for: Mailed Outreach for Colorectal Cancer Screening in Community Health Centers: The CARES Pragmatic Cluster Randomized Clinical Trial
Source: JAMA Intern Med. 2026 Apr 27;186(6):703–12. doi: 10.1001/jamainternmed.2026.1170 (PMC13122492; doi:10.1001/jamainternmed.2026.1170)
Supplement: Supplement 2. — eFigure 1. Time to screening participation over 180 days by study arm, among pragmatic, cluster randomized clinical trial participants (Boston and Los Angeles) eFigure 2. Time to screening participation by study arm from propensity score-matched sample of pragmatic, cluster randomized clinical trial participants (Boston and Los Angeles) eFigure 3. Time to screening participation among South Dakota participants (parallel arm) eFigure 4. Time to follow-up colonoscopy completion over 180 days among pragmatic, cluster randomized clinical trial participants (Boston and Los Angeles) with abnormal stool test results eTable 1. Population characteristics by site, including South Dakota eTable 2. Sensitivity analysis excluding participants who might have completed screening prior to receiving the screening kit among pragmatic, cluster randomized clinical trial participants (Boston and Los Angeles) eTable 3. Secondary analysis using the propensity score matched sample among pragmatic, cluster randomized clinical trial participants (Boston and Los Angeles) eTable 4. Primary and secondary outcomes of screening participation of South Dakota participants (parallel arm) eTable 5. Abnormal Screening Results by Study Arm (Per Protocol) vs. As Treated within 180 days, by region eTable 6. Navigation outcomes for patients with a positive stool test result (randomized clinical trial participants) eTable 7. Colonoscopy completion within 180 days after an abnormal stool test, pragmatic, cluster randomized clinical trial sites only eTable 8. Colonoscopy completion within 180 days after an abnormal stool test by region eTable 9. Exact Sciences Patient Navigation Outreach Data (within 45 days of FIT-DNA kit mailing) [file jamainternmed-e261170-s002.pdf]

Supplemental Online Content

May FP, Brodney S, Tuan JJ, et al. Mailed outreach for colorectal cancer screening in community health centers: the CARES pragmatic cluster randomized clinical trial. *JAMA Intern Med*. Published online April 27, 2026. doi:10.1001/jamainternmed.2026.1170

eFigure 1. Time to screening participation over 180 days by study arm, among pragmatic, cluster randomized clinical trial participants (Boston and Los Angeles)

eFigure 2. Time to screening participation by study arm from propensity score-matched sample of pragmatic, cluster randomized clinical trial participants (Boston and Los Angeles)

eFigure 3. Time to screening participation among South Dakota participants (parallel arm)

eFigure 4. Time to follow-up colonoscopy completion over 180 days among pragmatic, cluster randomized clinical trial participants (Boston and Los Angeles) with abnormal stool test results

eTable 1. Population characteristics by site, including South Dakota

eTable 2. Sensitivity analysis excluding participants who might have completed screening prior to receiving the screening kit among pragmatic, cluster randomized clinical trial participants (Boston and Los Angeles)

eTable 3. Secondary analysis using the propensity score matched sample among pragmatic, cluster randomized clinical trial participants (Boston and Los Angeles)

eTable 4. Primary and secondary outcomes of screening participation of South Dakota participants (parallel arm)

© 2026 May FP et al. *JAMA Intern Med*.

eTable 5. Abnormal Screening Results by Study Arm (Per Protocol) vs. As Treated within 180 days, by region

eTable 6. Navigation outcomes for patients with a positive stool test result (randomized clinical trial participants)

eTable 7. Colonoscopy completion within 180 days after an abnormal stool test, pragmatic, cluster randomized clinical trial sites only

eTable 8. Colonoscopy completion within 180 days after an abnormal stool test by region

eTable 9. Exact Sciences Patient Navigation Outreach Data (within 45 days of FIT-DNA kit mailing)

This supplemental material has been provided by the authors to give readers additional information about their work.

© 2026 May FP et al. *JAMA Intern Med*.

eFigure 1. Time to screening participation over 180 days by study arm, among pragmatic, cluster randomized clinical trial participants (Boston and Los Angeles).

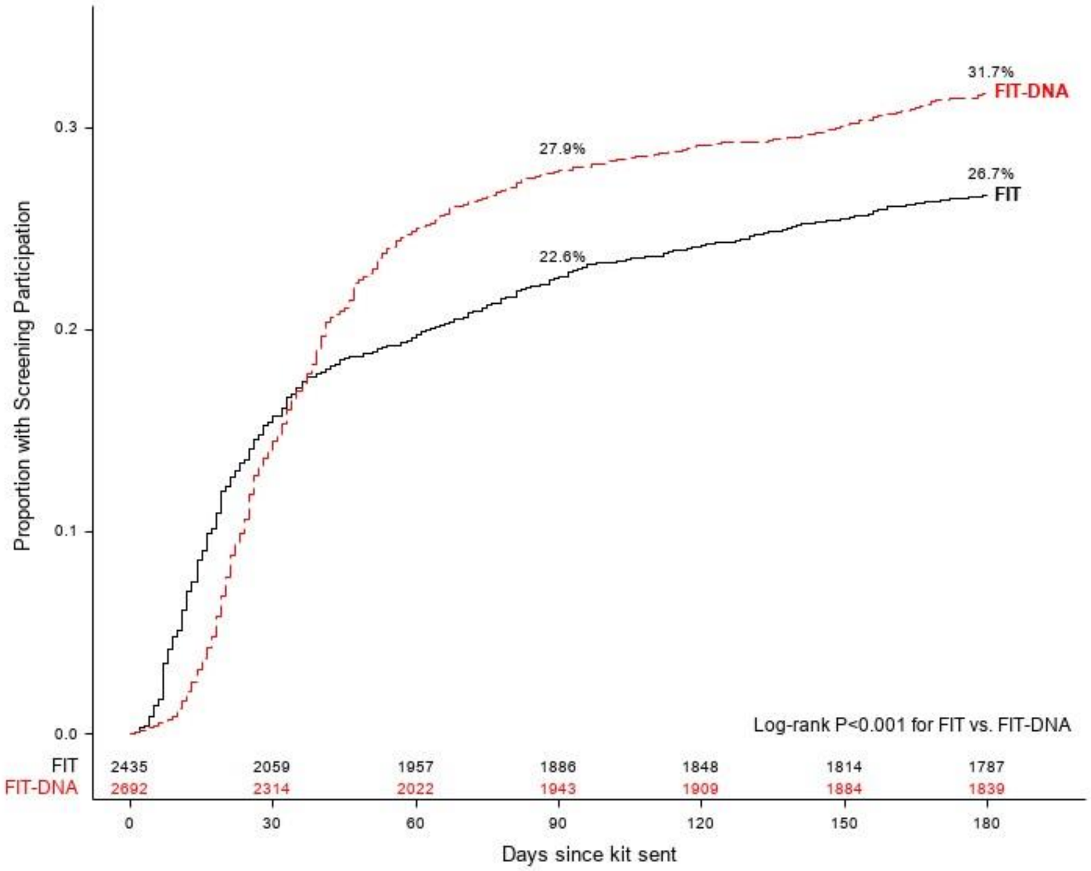

Figure Legend:
Abbreviations: FIT, fecal immunochemical test
Screening participation is defined as a test returned regardless of the result (e.g., normal, abnormal, inconclusive).
The p-value reflects the comparison of the groups randomized to FIT and FIT-DNA. X-axis displays the number at risk at each time point.

© 2026 May FP et al. *JAMA Intern Med*.

eFigure 2. Time to screening participation by study arm from propensity score-matched sample of pragmatic, cluster randomized clinical trial participants (Boston and Los Angeles).

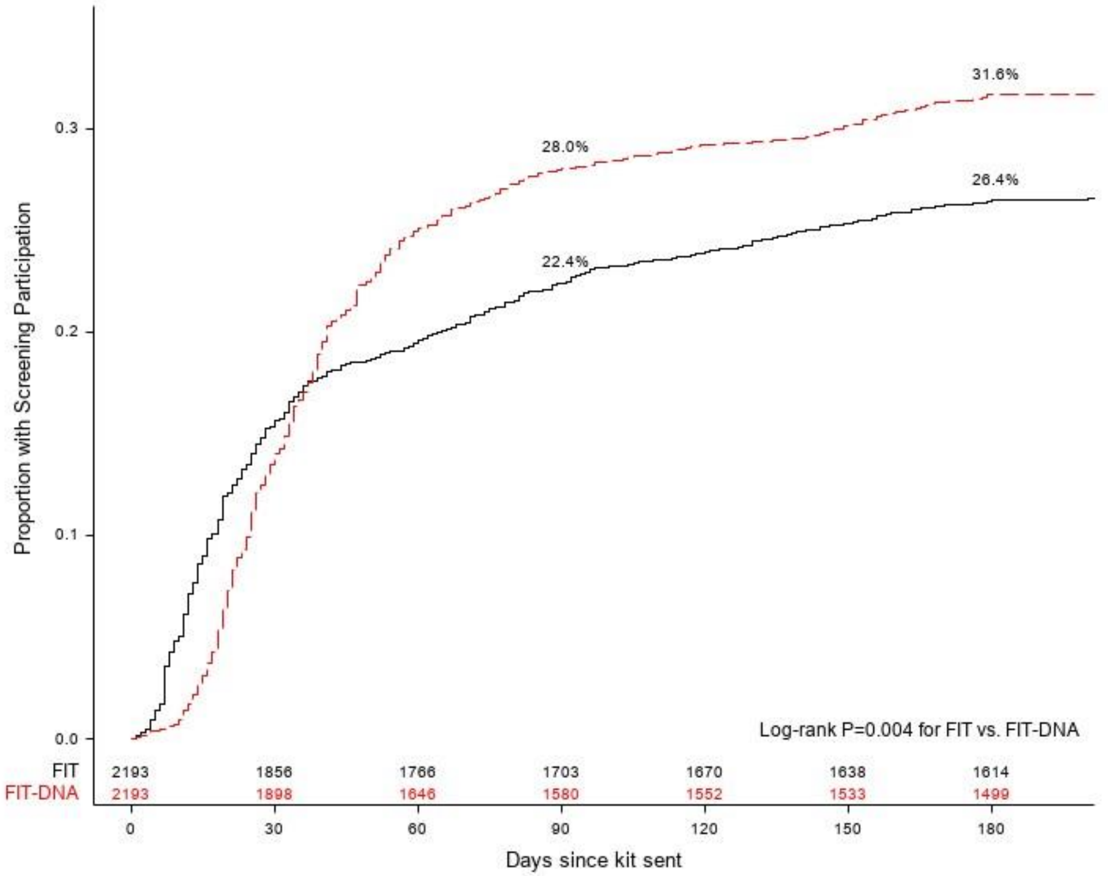

Figure Legend:
Abbreviations: FIT, fecal immunochemical test
Screening participation is defined as a test that is returned, regardless of the result (e.g., normal, abnormal, inconclusive).
The p-value reflects the comparison of the groups randomized to FIT and FIT-DNA. X-axis displays the number at risk at each time point.

© 2026 May FP et al. *JAMA Intern Med*.

eFigure 3. Time to screening participation among South Dakota participants (parallel arm).

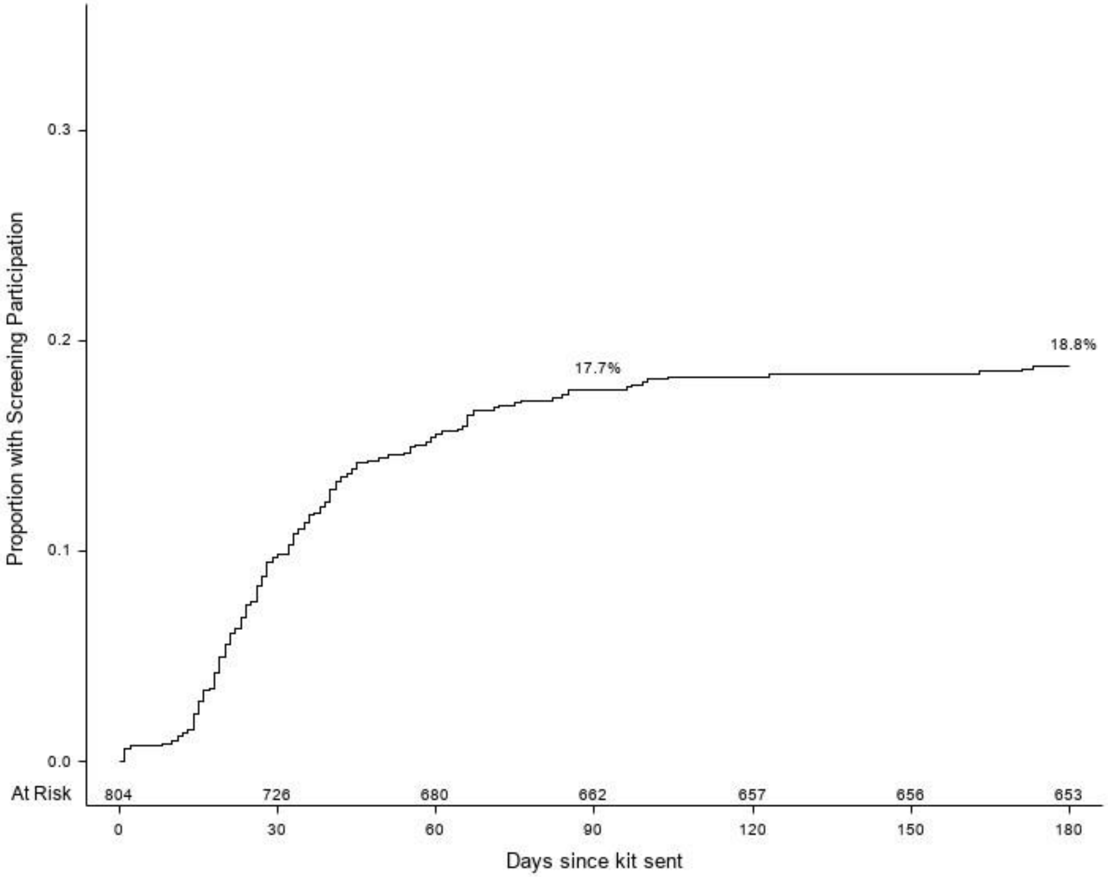

Figure Legend:
Screening participation is defined as a test returned regardless of the result (e.g., normal, abnormal, inconclusive).

© 2026 May FP et al. *JAMA Intern Med*.

eFigure 4. Time to follow-up colonoscopy completion over 180 days among pragmatic, cluster randomized clinical trial participants (Boston and Los Angeles) with abnormal stool test results.

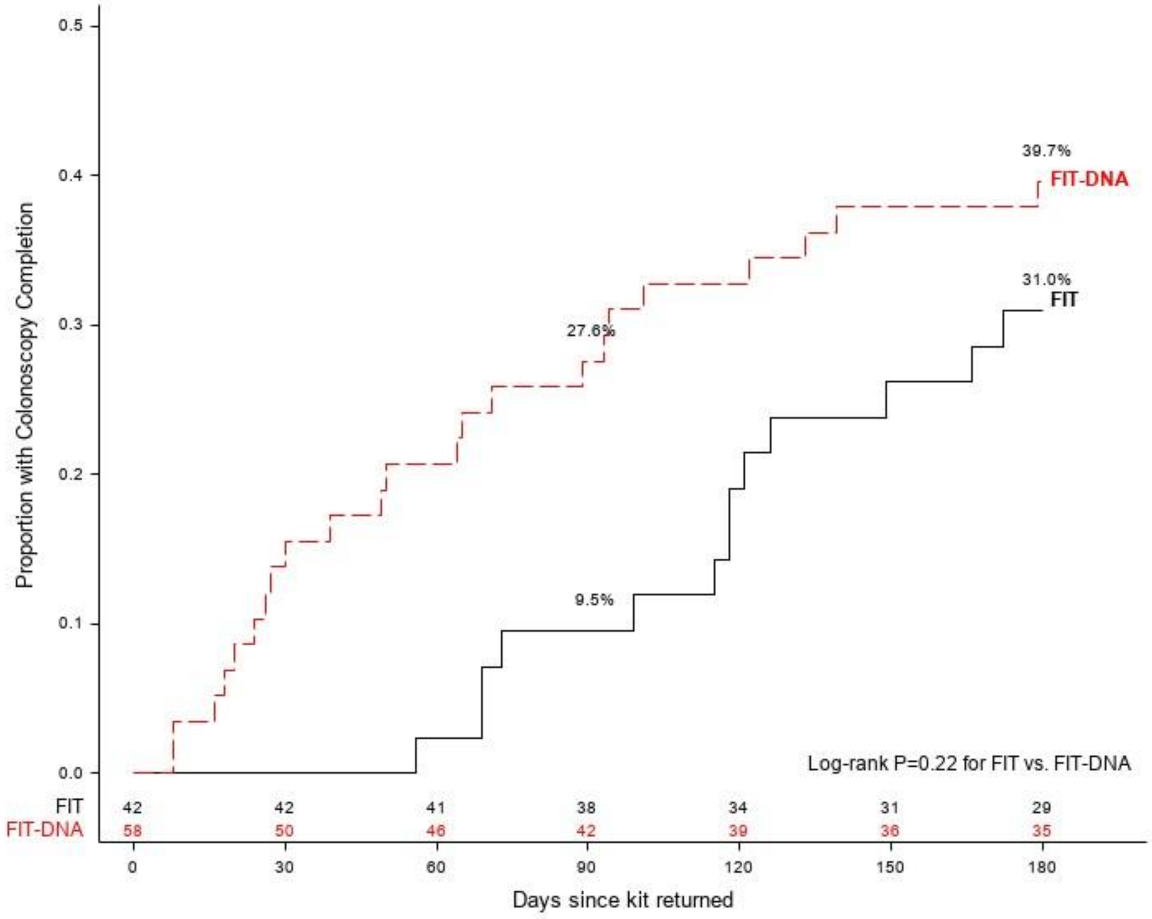

Figure Legend:
Abbreviations: FIT, fecal immunochemical test
The X-axis displays the number at risk at each time point. The p-value reflects the comparison of the randomized groups.

© 2026 May FP et al. *JAMA Intern Med*.

eTable 1. Population characteristics by site, including South Dakota.

	Randomized FIT arm				Randomized FIT-DNA arm				Non-Randomized Site
	Boston 1	Boston 2	LA 1	LA 2	Boston 1	Boston 2	LA 1	LA 2	South Dakota
**N**	483	467	752	733	792	466	712	722	804
**Age, y, mean (SD)**	53.0 (8.7)	53.5 (8.8)	55.8 (7.5)	56.0 (7.6)	52.1 (8.3)	53.0 (8.8)	56.1 (7.5)	55.5 (7.5)	59.9 (6.9)
**Age category, y, n (%)**									
< 50 [a]	262 (56.1)	300 (62.1)	170 (23.2)	178 (23.7)	274 (58.8)	500 (63.1)	186 (25.8)	171 (24.0)	0
≥ 50	205 (43.9)	183 (37.9)	563 (76.8)	574 (76.3)	192 (41.2)	292 (36.9)	536 (74.2)	541 (76.0)	804 (100.0)
**Sex, n (%)**									
Female	319 (68.3)	250 (51.8)	408 (55.7)	460 (61.2)	268 (57.5)	459 (58.0)	437 (60.5)	417 (58.6)	447 (55.6)
Male	148 (31.7)	233 (48.2)	325 (44.3)	292 (38.8)	198 (42.5)	333 (42.0)	285 (39.5)	295 (41.4)	357 (44.4)
**Race/Ethnicity, n (%)**									
Non-Hispanic, White	53 (11.3)	378 (78.3)	15 (2.0)	10 (1.3)	113 (24.2)	166 (21.0)	12 (1.7)	16 (2.2)	0
Non-Hispanic, Black	40 (8.6)	35 (7.2)	106 (14.5)	15 (2.0)	51 (10.9)	54 (6.8)	23 (3.2)	45 (6.3)	0
Hispanic	352 (75.4)	44 (9.1)	602 (82.1)	724 (96.3)	268 (57.5)	506 (63.9)	680 (94.2)	642 (90.2)	0
American Indian	0	0	0	0	0	0	0	0	804 (100)
Other[b]/Unknown	22 (4.7)	26 (5.4)	10 (1.4)	3 (0.4)	34 (7.3)	66 (8.3)	7 (1.0)	9 (1.3)	0
**Language, n (%)**									
English	212 (45.4)	461 (95.4)	170 (23.2)	64 (8.5)	281 (60.3)	373 (47.1)	101 (14.0)	102 (14.3)	

© 2026 May FP et al. *JAMA Intern Med*.

Spanish	255 (54.6)	22 (4.6)	563 (76.8)	688 (91.5)	185 (39.7)	419 (52.9)	621 (86.0)	610 (85.7)	
**Insurance Type, n (%)**									
Medicare	45 (9.6)	75 (15.5)	47 (6.4)	56 (7.4)	58 (12.4)	85 (10.7)	33 (4.6)	42 (5.9)	0
Medicaid	148 (31.7)	35 (7.2)	505 (68.9)	532 (70.7)	83 (17.8)	209 (26.4)	487 (67.5)	541 (76.0)	0
Private	257 (55.0)	355 (73.5)	40 (5.5)	30 (4.0)	299 (64.2)	457 (57.7)	28 (3.9)	48 (6.7)	0
Uninsured	17 (3.6)	17 (3.5)	135 (18.4)	133 (17.7)	24 (5.2)	39 (4.9)	172 (23.8)	77 (10.8)	0
Indian Health Service	0	0	0	0	0	0	0	0	804 (100)
Other[c]	0	1 (0.2)	6 (0.8)	1 (0.1)	2 (0.4)	2 (0.3)	2 (0.3)	4 (0.6)	0

Abbreviations:
LA, Los Angeles
FIT, fecal immunochemical test
Data Source: Electronic health record (EHR) data.
NOTES:
[a] Screening initiated at age 50 in South Dakota during the study period
[b] Other includes Non-Hispanic Asian (n=47), Pacific Islander (n=1), American Indian (n=7), and multiple races (n=3). American Indian is not displayed separately for the randomized sites because of small cell sizes.
[c] Other includes payors that may not have covered CRC screening but were listed in the EHR as the primary insurer, including: vision insurance, workers' compensation, motor vehicle insurance, family planning, Ryan White, and country- or locally sponsored health services.

.

© 2026 May FP et al. *JAMA Intern Med*.

eTable 2. Sensitivity analysis excluding participants who might have completed screening prior to receiving the screening kit among pragmatic, cluster randomized clinical trial participants (Boston and Los Angeles).

	**FIT (N=2,350)**	**FIT-DNA (N=2,677)**	**Adjusted Difference [a], % (95% CI)**
**Screening participation within 90 days, n (%)**	465 (19.8)	736 (27.5)	7.1 (3.0-11.3)
**Screening participation within 180 days, n (%)**	564 (24.0)	839 (31.3)	6.8 (2.6-11.0)

Abbreviations: FIT, fecal immunochemical test; CI, confidence interval

NOTES:

© 2026 May FP et al. *JAMA Intern Med*.

eTable 3. Secondary analysis using the propensity score matched sample among pragmatic, cluster randomized clinical trial participants (Boston and Los Angles).

	FIT (N=2,193)	FIT-DNA (N=2,193)	Adjusted Difference[a], % (95% CI)
**Participation within 90 days, n (%)**	491 (22.4)	615 (28.0)	5.3 (1.2-9.5)
**Screening participation within 180 days, n (%)**	580 (26.4)	694 (31.6)	5.0 (1.0-9.0)

© 2026 May FP et al. *JAMA Intern Med*.

eTable 4. Primary and secondary outcomes of screening participation of South Dakota participants (parallel arm).

	**FIT-DNA**
	**N=804**
**Screening participation within 90 days, n (%)**	142 (17.7)
**Screening participation within 180 days, n (%)**	151 (18.8)

Abbreviations: FIT, fecal immunochemical test

© 2026 May FP et al. *JAMA Intern Med*.

eTable 5. Abnormal Screening Results by Study Arm (Per Protocol) vs. As Treated within 180 days, by region.

	Boston			LA			South Dakota		
	**Total (N=663)**	**Per Protocol (randomized)**		**Total (N=772)**	**Per Protocol (randomized)**		**Total (N=149)**	**Parallel Arm**	
		**FIT (N=291)**	**FIT-DNA (N=372)**		**FIT (N=332)**	**FIT-DNA (N=440)**		**FIT (N=0)**	**FIT-DNA (N=149)**
**Screening test completed**									
FIT	312	284	28	418	332	86	19	NA	19
FIT-DNA	351	7	344	354	0	354	130		130
**Per protocol**	**Total (N=663)**	**FIT (N=291)**	**FIT-DNA (N=372)**	**Total (N=772)**	**FIT (N=332)**	**FIT-DNA (N=440)**	**Total (N=149)**	**FIT (N=0)**	**FIT-DNA (N=149)**
Negative, n (%)	605 (91.3)	278 (95.5)	327 (87.9)	635 (82.3)	245 (73.8)	390 (88.6)	116 (77.9)	NA	116 (77.9)
Positive, n (%)	33 (5.0)	6 (2.1)	27 (7.3)	67 (8.7)	36 (10.8)	31 (7.0)	26 (17.4)		26 (17.4)
Inconclusive, n (%)	25 (3.8)	7 (2.4)	18 (4.8)	70 (9.1)	51 (15.4)	19 (4.3)	7 (4.7)		7 (4.7)
**As treated**	**Total (N=663)**	**FIT (N=312)**	**FIT-DNA (N=351)**	**Total (N=772)**	**FIT (N=418)**	**FIT-DNA (N=354)**	**Total (N=149)**	**FIT (N=19)**	**FIT-DNA (N=130)**
Negative, n (%)	605 (91.3)	298 (95.5)	307 (87.5)	635 (82.3)	323 (77.3)	312 (88.1)	116 (77.9)	8 (42.1)	108 (83.1)
Positive, n (%)	33 (5.0)	7 (2.2)	26 (7.4)	67 (8.7)	43 (10.3)	24 (6.8)	26 (17.4)	11 (57.9)	15 (11.5)
Inconclusive, n (%)	25 (3.8)	7 (2.2)	18 (5.1)	70 (9.1)	52 (12.4)	18 (5.1)	7 (4.7)	0	7 (5.4)

Abbreviations: FIT, fecal immunochemical test

© 2026 May FP et al. *JAMA Intern Med*.

eTable 6. Navigation outcomes for patients with a positive stool test result (randomized clinical trial participants).

	All			Boston			Los Angeles		
	**All**	**FIT**	**FIT-DNA**	**All**	**FIT**	**FIT-DNA**	**All**	**FIT**	**FIT-DNA**
**Never reached**	37	11 (29.7)	26 (70.3)	3	1 (33.3)	2 (66.6)	34	10 (29.4)	24 (70.6)
**Refused**	4	0 (0.0)	4 (100.0)	2	0 (0.0)	2 (100.0)	2	0 (0.0)	2 (100.0)
**Received navigation**	59	31 (52.5)	28 (47.5)	28	5 (21.7)	23 (82.4)	31	26 (83.9)	5 (16.1)
**Total**	100	42 (42.0)	58 (58.0)	33	6 (18.2)	27 (81.8)	67	36 (53.7)	31 (46.3)

Abbreviations: FIT, fecal immunochemical test.

© 2026 May FP et al. *JAMA Intern Med*.

eTable 7. Colonoscopy completion within 180 days after an abnormal stool test, randomized clinical trial sites only (randomized clinical trial participants).

	**Total (N=100)**	**Per Protocol**	
		**FIT (N=42)**	**FIT-DNA (N=58)**
**Screening test completed**			
FIT	50	42	8
FIT-DNA	50	0	50
**Per protocol**	**Total (N=100)**	**FIT (N=42)**	**FIT-DNA (N=58)**
Completed, n (%)	36 (36.0)	13 (31.0)	23 (39.7)
**As treated**	**Total (N=100)**	**FIT (N=50)**	**FIT-DNA (N=50)**
Completed, n (%)	36 (36.0)	14 (28.0)	22 (44.0)
Abbreviations: FIT, fecal immunochemical test.			

© 2026 May FP et al. *JAMA Intern Med*.

eTable 8. Colonoscopy completion within 180 days after an abnormal stool test by region.

	Boston			Los Angeles			South Dakoda		
	Total (N=33)	Per Protocol (randomized)		Total (N=67)	Per Protocol (randomized)		Total (N=26)	Parallel Arm	
		FIT (N=36)	FIT-DNA (N=31)		FIT (N=36)	FIT-DNA (N=31)		FIT (N=0)	FIT-DNA (N=26)
**Screening test completed**									
FIT	7	6	1	43	36	7	11	NA	11
FIT-DNA	26	0	26	24	0	24	15		15
**Per protocol**	**Total (N=33)**	**FIT (N=36)**	**FIT-DNA (N=31)**	**Total (N=67)**	**FIT (N=36)**	**FIT-DNA (N=31)**	**Total (N=26)**	**FIT (N=0)**	**FIT-DNA (N=26)**
Completed, n (%)	23 (69.7)	4 (66.7)	19 (70.4)	13 (19.4)	9 (25.0)	4 (12.9)	12 (46.2)	0	12 (46.2)
**As treated**	**Total (N=33)**	**FIT (N=7)**	**FIT-DNA (N=26)**	**Total (N=67)**	**FIT (N=43)**	**FIT-DNA (N=24)**	**Total (N=26)**	**FIT (N=11)**	**FIT-DNA**
Completed, n (%)	23 (69.7)	18 (69.2)	5 (71.4)	13 (19.4)	9 (20.9)	4 (16.7)	12 (46.2)	3 (27.3)	9 (60.0)

Abbreviations: FIT, fecal immunochemical test.

© 2026 May FP et al. *JAMA Intern Med*.

eTable 9. Exact Sciences Patient Navigation Outreach Data (within 45 days of FIT-DNA kit mailing).

	Share of patients with outreach	Outreach count - Median	Outreach count - 10th Percentile [a]	Outreach count - 90th Percentile [a]
**Total Outreach**	99.7%	10	5	11
**Outreach by Return Status**				
Not Returned	99.9%	10	7	12
Returned	99.0%	8	4	11
**Outreach by Modality**				
Text	94.0%	6	2	7
Email[b]	4.8%	0	0	0
Call	97.4%	2	1	4
Letter	99.5%	2	1	2
**Outreach by Modality and Return Status**				
Text				
Not Returned	94.0%	7	2	7
Returned	93.0%	4	1	7
Email[b]				
Not Returned	4.0%	0	0	0
Returned	6.0%	0	0	0
Call				
Not Returned	97.0%	2	1	4
Returned	98.0%	2	1	3
Letter				
Not Returned	100.0%	2	2	2
Returned	99.0%	2	1	2

[a] The data provided represent the 10th and 90th percentile of subjects to exclude the small number of cases in which outreach failed due to text messages to landlines and telephone lines that blocked unrecognized numbers.

[b] The small number of email outreaches (4.8%) reflects the fact that Exact Sciences laboratory had email addresses for a very limited subset of patients.

© 2026 May FP et al. *JAMA Intern Med*.
