# Supplementary material for: Mailed Outreach for Colorectal Cancer Screening in Community Health Centers: The CARES Pragmatic Cluster Randomized Clinical Trial
Source: JAMA Intern Med. 2026 Apr 27;186(6):703–12. doi: 10.1001/jamainternmed.2026.1170 (PMC13122492; doi:10.1001/jamainternmed.2026.1170)
Supplement: Supplement 3. — Data Sharing Statement [file jamainternmed-e261170-s003.pdf]

Data Sharing Statement

May. Mailed Outreach for Colorectal Cancer Screening in Community Health Centers. *JAMA Intern Med*. Published April 27, 2026. doi:10.1001/jamainternmed.2026.1170

Data
Additional Information: ClinicalTrials.gov Identifier: NCT05714644 (https://register.clinicaltrials.gov/prs/beta/studies/S000CLWX00000090/recordSummary). Date of registration: 1/27/2023
Data available: No

Additional Information
Explanation for why data not available: Requests for data or study materials can be made to the corresponding author [JSH] and will be considered based on available resources and data sharing policies.
